# *Mycobacterium tuberculosis* infection triggers IL-1β-dependent epigenetic training in bystander macrophages

**DOI:** 10.1128/mbio.01214-26

**Published:** 2026-07-21

**Authors:** Shah-e-Jahan Gulzar, Ibrahim Umar, Bharath Saravanan, Dhruv Sheth, Arif Hussain Najar, Umer Farooq, Sahanawaz Molla, Adithi Ravikumar, Dimple Notani, Varadharajan Sundaramurthy

**Affiliations:** 1 National Centre for Biological Sciences, Bangalore, India; 2 SASTRA University 93161, Thanjavur, India; Washington University in St. Louis, St. Louis, Missouri, USA

**Keywords:** tuberculosis, bystander cells, IL-1β, trained immunity, epigenetic modifications, H3K27 acetylation, superinfection

## Abstract

*Mycobacterium tuberculosis* (*Mtb*) remodels host cell functions to support its persistence within macrophages. While infected cells have been extensively studied, the responses of uninfected bystander macrophages in the same microenvironment remain poorly understood. Here, we demonstrate that *Mtb* infection triggers broad epigenetic and transcriptional reprogramming in bystander macrophages, predominantly via interleukin-1β-dependent nuclear factor-κB signaling from infected cells. These bystander cells acquire active chromatin marks, exhibit distinct gene expression profiles, and display enhanced responsiveness to subsequent immune challenges. Functionally, bystander macrophages restrict intracellular *Mtb* growth and also show increased responsiveness to heterologous stimuli resembling trained immunity. Our findings uncover a previously underappreciated mechanism of intercellular communication during infection, wherein *Mtb*-infected macrophages prime neighboring uninfected cells for enhanced defense. This work defines cytokine-mediated reprogramming of both infected and bystander cell subpopulations, and identifies bystander cells as active participants in shaping the population-wide host immune landscape. These insights have implications for understanding innate immune memory and developing strategies to modulate host defense in tuberculosis and other infections.

**IMPORTANCE:**

This study reveals an underappreciated role for uninfected bystander macrophages in host defense against *Mycobacterium tuberculosis* (*Mtb*). We demonstrate that *Mtb*-infected macrophages trigger interleukin-1β-mediated epigenetic training in neighboring bystander cells, priming them for enhanced immune responses. These trained macrophages exhibit heightened antimicrobial activity and restrict *Mtb* growth upon subsequent infection. By uncovering a mechanism through which immune memory-like responses propagate beyond infected cells, our findings redefine the cellular scope of innate immunity during tuberculosis and identify new opportunities to boost host defense through intercellular signaling and epigenetic reprogramming.

## INTRODUCTION

*Mycobacterium tuberculosis* (*Mtb*), the causative agent of tuberculosis (TB), is often referred to as the “world’s most successful pathogen” due to its remarkable ability to manipulate host cell functions. Recent studies have increasingly highlighted that *Mtb* not only alters the function of the infected cell but also adjacent uninfected “bystander” cells. For example, bystander cells exhibit enhanced apoptosis, impaired dendritic cell maturation, increased HIV transmission, and metabolic shifts, including mitochondrial fragmentation and glycolysis (1–4). Furthermore, distinct gene expression patterns triggered by *Mtb* infection have been identified in distal, circulating blood cells (5, 6), suggesting systemic effects beyond the localized infection site. Indeed, single-cell transcriptomic analyses from *Mtb*-infected murine lungs revealed substantial transcriptional alterations in bystander cells (7) compared to naive cells. Additionally, sorted populations from infected lungs suggest that infected and bystander cells of the same type cluster together by gene expression profile (8). Bystander effects may be mediated via exposure of bystander cells to mycobacterial lipids, pathogen-associated molecular patterns (9, 10), antigen transfer (11), or infection-induced soluble mediators such as chemokines and cytokines (12). Interestingly, *Mtb-*derived lipids have been detected in bystander cells as well as in distal sites of infected animals (9, 13), suggesting direct influence of *Mtb* beyond the infected cells. Despite this growing body of evidence, the extent and relevance of the modulation of bystander cell functions during *Mtb* infection, and the underlying mechanisms, remain poorly understood.

It is well known that *Mtb* infection alters the transcriptional landscape of infected cells, largely through epigenetic modifications (14–16). *Mtb* interferes with host epigenetic machinery by altering specific histone modifications such as methylation, acetylation, and phosphorylation, as well as directly affecting DNA methylation (17). Specific *Mtb* factors actively contribute to host epigenetic reprogramming. Rv1988, a histone methyltransferase, specifically represses NADPH oxidase, nitric oxide synthase (NOS2), and other immune-related genes (18); Rv2067c trimethylates H3K79 in the non-tail region to promote its survival (19); Rv2966c, a 5-methylcytosine DNA methyltransferase, is involved in non-CpG methylation (20), while Rv3423.1, a secreted protein, and Rv0428, expressed in the phagosome, demonstrate *in vitro* histone acetyl transferase (HAT) activity (21, 22). In addition, well-studied *Mtb* products like ESAT-6 and ManLAM also contribute to epigenetic changes (23). A significant consequence of these alterations is the direct modulation of crucial immune pathways, such as inflammation and immune responses, through key mediators like the NLRP3 inflammasome and TLR signaling (17, 24). Beyond these gene-specific effects, emerging reports suggest genome-scale epigenetic reprogramming during mycobacterial infections. Whole-genome methylation profiling in pulmonary TB patients reveals differentially methylated loci, primarily localized to the autophagic pathway, that change upon anti-TB drug treatment, highlighting the specificity and dynamism of these changes (24). Conversely, another clinical study proposes global DNA hypomethylation in pediatric TB patients (25). These studies, along with evidence of systemic epigenetic changes (6, 26) in TB patients, underscore both large-scale remodeling of the host epigenetic landscape by *Mtb* as well as its far-reaching effects on cells and tissues beyond the immediate site of infection. Given these profound changes in infected macrophages, it is plausible that neighboring bystander cells might undergo similar epigenetic remodeling, yet this remains underexplored.

Trained immunity (TI) is an emerging concept in immunology where innate immune cells such as macrophages exhibit a “memory-like” property (27–29), by showing a heightened immune response to a secondary stimulus following “training” by a primary stimulus. These changes are driven by epigenetic and metabolic alterations induced by the primary stimulus. Interestingly, *Mycobacterium bovis* BCG and *Mtb* are commonly used as primary stimuli in many TI experimental paradigms (29, 30). However, the precise mechanisms by which mycobacterial-induced training propagates to the infected population that includes infected and bystander subpopulations are not clear. Here, we show that mycobacterial infection triggers H3K27Ac enrichment in both infected and neighboring bystander macrophages, leading to transcriptional changes associated with trained immunity. Mechanistically, we identify IL-1β as a key mediator of this intercellular epigenetic communication. Functionally, bystander macrophages exhibit enhanced responsiveness and restrict subsequent infections, including *Mtb*. Our findings reveal that *Mtb* infection propagates a population-wide immune training via cytokine-mediated epigenetic reprogramming, implicating bystander cells as active contributors to host defense.

## RESULTS

### Bystander cells restrict *Mtb* growth upon reinfection

**Fig 1:**
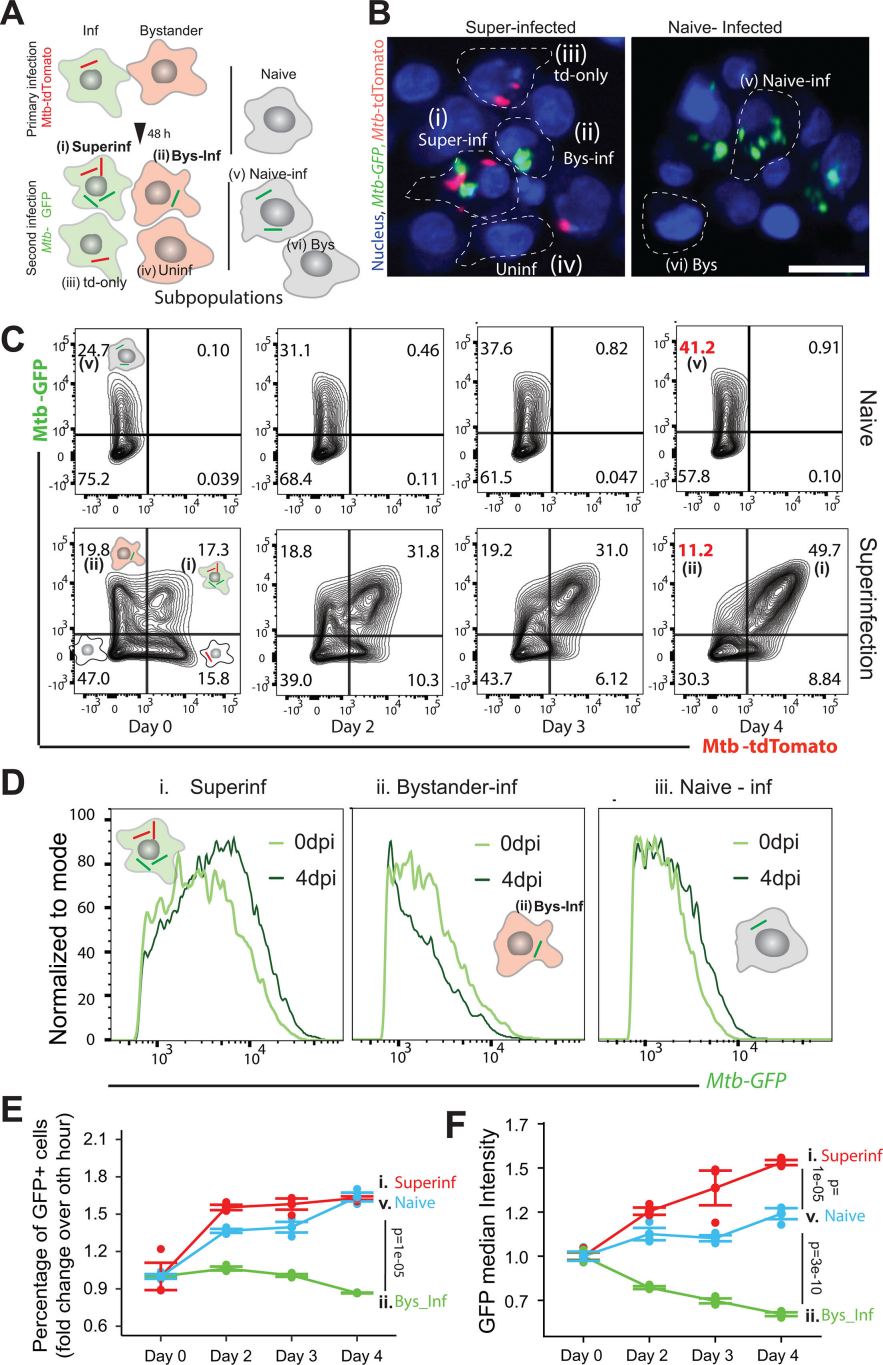
Bystander cells restrict intracellular *Mtb* growth. (**A**) Scheme of the experiment. (**B**) Confocal images of THP-1 MDMs infected with tdTomato-tagged H37Rv for 48 h, followed by superinfection with *Mtb*-GFP H37Rv. After 4 h of uptake of the second bacteria, cells were fixed and imaged. Scale bar is 25 μm. (**C**) Representative flow cytometry contour plots showing *Mtb*-GFP H37Rv (*y*-axis) versus *Mtb*-tdTomato H37Rv (primary infection, *x*-axis) fluorescence over time (day 0–day 4). Cells were first gated for singlets. Within the single-cell population, a gate was applied to distinguish naive, Bys-Inf, and Inf-Inf cells based on *Mtb*-tdTomato and *Mtb*-GFP expression. GFP expression was quantified from this gated population. Numbers in the quadrants indicate the percentages of the respective subpopulations. (**D**) Density plot overlays displaying the shift in the fluorescent signal of intracellular *Mtb-*GFP intensities in the indicated subpopulations between day 0 and day 4. Day 0 represents bacterial uptake immediately following the second infection. Data are from 50,000 cells. (**E and F**) Kinetics of the secondary infection (*Mtb*-GFP H37Rv) in the three subpopulations (only GFP+ or naive, tdTomato−/GFP+ or Bys-Inf, and tdTomato+/GFP+ or Inf-Inf) normalized to the *Mtb*-GFP median intensity or percentage of GFP+ cells at day 0/uptake. Data represent mean ± SEM of three technical replicates. Statistical significance was determined by one-way ANOVA with Tukey’s multiple comparisons test, and exact *P*-values are indicated in the figure. Results are representative of at least three independent experiments.

Non-infected cells within an infected population, referred to as bystander cells, have traditionally been considered passive participants in infection outcomes. However, emerging evidence indicates that bystander cells are transcriptionally distinct from naive cells (7, 8), suggesting they may respond differently to subsequent infections. To investigate this, we first infected THP-1 monocyte-derived macrophages (THP-1 MDMs) with *Mycobacterium tuberculosis* expressing tdTomato. Forty-eight hours later, the same population was reinfected with *Mtb* expressing GFP (Fig. 1A), generating four distinct subpopulations: (i) cells harboring both *Mtb*-tdTomato and *Mtb*-GFP (tdT+, GFP+; superinfected), (ii) cells infected only with *Mtb*-GFP (tdT−, GFP+; bystander-infected), (iii) cells infected only with *Mtb*-tdTomato (tdT+, GFP−) and not in the second round, and (iv) cells that remained uninfected in both rounds of infection (tdT−, GFP−). As a control for this assay, naive cells were infected with *Mtb*-GFP, and the resulting *Mtb*-GFP-positive cells were classified as naive-infected. Microscopy images (Fig. 1B) show the resulting cellular subpopulations. The subpopulations of particular interest are i and ii. To test if bystander cells respond differently to subsequent infection, we compared the progression of the second infection, *Mtb*-GFP, across these subpopulations by flow cytometry (gating strategy in Fig. S1 at https://doi.org/10.6084/m9.figshare.32879825). Quantitative analysis (Fig. 1D and F) revealed that intracellular *Mtb*-GFP fluorescence intensity increased from 0 to 4 days post-infection in both naive-infected and superinfected cells but surprisingly, decreased in bystander-infected cells. A similar trend was observed in the proportion of *Mtb*-GFP-positive cells in naive-infected versus bystander-infected populations (Fig. 1C and E). These findings show that bystander cells actively restrict intracellular *Mtb* growth upon reinfection.

To further test the robustness of the growth-restrictive phenotype of the bystander cells, we examined the progression of second-infected *Mtb* at varying bacterial loads (see Fig. S2A and B at https://doi.org/10.6084/m9.figshare.32879825). Flow cytometry analysis at both low and high multiplicity of infection (MOI) showed that the *Mtb*-GFP fluorescence intensity remained consistently lower in bystander-infected cells than in naive-infected and superinfected cells, demonstrating that the restrictive phenotype of bystander cells is sustained even under increased infectious pressure. To exclude the possibility that GFP quenching within acidified compartments could result in false-negative classification in the bystander cells, we reversed the order of the labeled *Mtb* during infection (Fig. S2C) and assessed the intracellular proliferation of *Mtb*-tdTomato in the second round. The results (Fig. S2D through G) show a similar restriction of the second *Mtb* infection in bystander cells.

To determine whether the enhanced bacterial burden observed in superinfected cells compared to the bystander cells could be associated with increased cell death and associated loss of intracellular control (31–33), we performed cell-death analysis in the different subpopulations following the second round of infection. The results (Fig. S2H and I) show higher cell death in the superinfected subpopulation, suggesting that these cells are on the path to cell death, potentially explaining the uncontrolled progression of the secondary infection. Collectively, these results demonstrate that subpopulations of cells in the infection milieu respond distinctly to a subsequent infection, with bystander macrophages actively restricting *Mtb* growth upon reinfection.

### Epigenetics-mediated transcriptional changes in bystander cells

**Fig 2:**
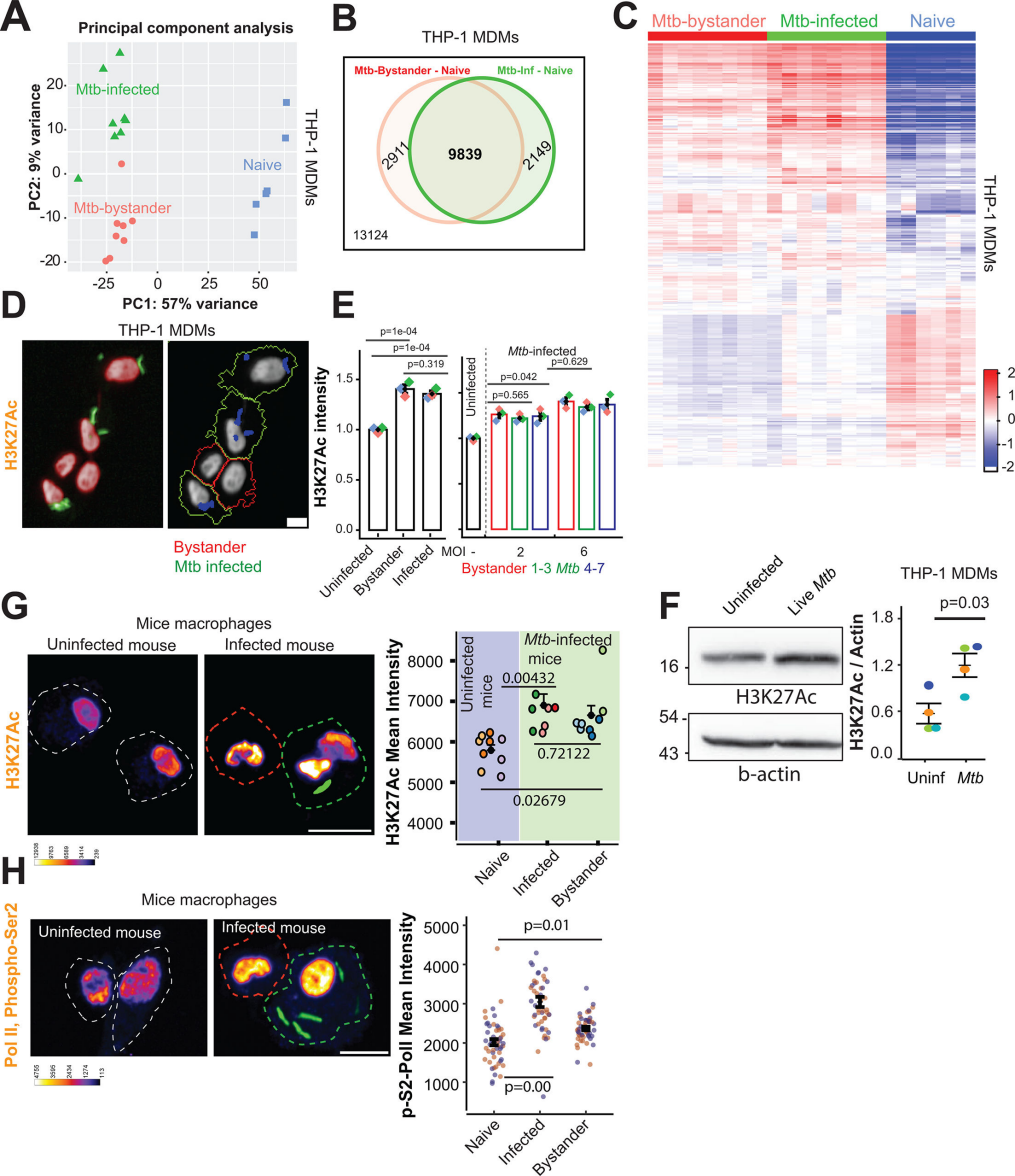
Gene expression alterations in bystander cells correlate with H3K27Ac status. (**A**) PCA of gene expression data from naive, *Mtb*-infected, and *Mtb*-bystander THP-1 MDM. (**B**) Venn diagram showing the number of differentially expressed genes (log2 fold change ≥ 1.5) relative to naive cells across the indicated conditions. (**C**) Heatmap showing expression levels of the top 400 variable genes between *Mtb*-bystander, *Mtb*-infected, and naive THP-1 MDM. (**D**) Immunostaining of H3K27Ac in THP-1 MDMs infected with *Mtb*-mCherry. The right panel shows the segmentation of the infected and bystander subpopulations based on the *Mtb* presence. Each dot represents an independent biological replicate. Data are mean ± SEM (*n* = 3). (**E**) Quantification of H3K27Ac intensity in nuclei of cells classified as low load (one to three bacilli, green), high load (four to six bacilli, blue), and bystander cells (red) based on the number of *Mtb* per cell. Each dot represents an independent biological replicate. Data are mean ± SEM (*n* = 3). (**F**) Representative western blot and densitometry of H3K27Ac of THP-1 MDMs infected with *Mtb* mCherry for 48 h; beta-actin was used as a loading control. Individual biological replicates are color-coded in the dot plot. Statistical significance was determined using Student’s *t*-test; error bars indicate mean ± SEM. (**G**) Macrophages isolated from the lungs of Mtb-GFP-infected C3HeB/FeJ mice were immunostained for H3K27Ac. Mean fluorescence intensity (MFI) was quantified across naive, infected, and bystander populations. Each data point represents an individual technical replicate, with colors indicating individual mice. (**H**) Macrophages isolated from the lungs of *Mtb* GFP-infected C3HeB/FeJ mice were immunostained for H3K27Ac (**G**) or Pol II phospho-Ser2 (**H**). MFI was quantified across populations. Each data point represents an individual image field, with colors indicating individual mice. Statistical significance was assessed using one-way ANOVA with Tukey’s multiple comparisons test. For panels **D**, **G, **and **H**, the scale bar is 10 μm. Dashed lines in** panels D**, **G**, and **H** denote cell boundaries. Gene expression data set for panels **A–C** is from Jani et al. (34).

To explore the mechanism of differential control in the infected and bystander subpopulations, we analyzed an RNA-seq data set of naive THP-1 macrophages as well as *Mtb*-infected and bystander subpopulations (34). Principal component analysis (PCA) showed that *Mtb*-infected and bystander cells were closely clustered along the first principal component, while both were distinct from naive cells (Fig. 2A), suggesting a strong influence of *Mtb* infection on bystander cell transcription. A substantial number of differentially expressed genes showed similarity in gene expression between *Mtb*-infected and bystander cells relative to naive controls (Fig. 2B), as also reflected in the heat map of the top 400 variable genes (Fig. 2C). These data indicate a striking difference in gene expression between naive and bystander macrophages, highlighting the impact *Mtb* infection has on the bystander subpopulations.

Epigenetic alterations often underlie gene expression changes in diverse contexts. The two most significant and well-understood regulatory regions for gene transcription, namely active promoters and distal enhancers, are both marked by H3K27Ac (6). It dynamically marks the genome upon development and signaling cues to regulate precise gene transcriptional outputs (35). We asked if mycobacterial infection alters H3K27Ac levels in bystander cells. Toward this, we immunostained the *Mtb-*infected cell population with an antibody specific to H3K27Ac and compared the signal intensities between the infected and bystander subpopulations with naive cells. Quantitative analysis (Fig. 2D) revealed no significant difference in H3K27Ac levels between these two subpopulations, while importantly, both exhibited significantly elevated levels compared to naive cells. Importantly, this increase required live Mycobacteria infection, as heat-killed *Mtb* did not induce H3K27Ac enrichment (see Fig. S3B at https://doi.org/10.6084/m9.figshare.32879825), indicating that active Mycobacteria-macrophage interactions are necessary for this epigenetic response. We further computationally stratified infected cells based on intracellular *Mtb* burden and analyzed H3K27Ac levels across the different infection bins. Notably, the overall population showed increased H3K27Ac with higher multiplicity of infection, independent of individual cellular *Mtb* load (Fig. 2E). This enhancement in H3K27Ac was reproducible across different mycobacterial infection models, including primary macrophages and both *Mtb* and *M. bovis* BCG infections (Fig. S3A and C), indicating a conserved host response across mycobacterial species. Western blotting further corroborated the infection-induced increase in H3K27Ac (Fig. 2F). Finally, to assess if increased H3K27Ac levels are observed during *in vivo* infection, we isolated macrophages from the lungs of *Mtb*-infected mice and immunostained for H3K27Ac. The results show that both infected and bystander cells displayed elevated H3K27Ac levels (Fig. 2G) compared to naive macrophages. Consistently, phospho-Ser2 RNA polymerase II—a marker of active transcription—was similarly enriched in both subpopulations compared to macrophages from uninfected animals (Fig. 2H). Together, these results show gene expression changes in *Mtb*-bystander cells (Fig. 2A and B), possibly mediated by epigenetic alterations in these subpopulations (Fig. 2D).

**Fig 3:**
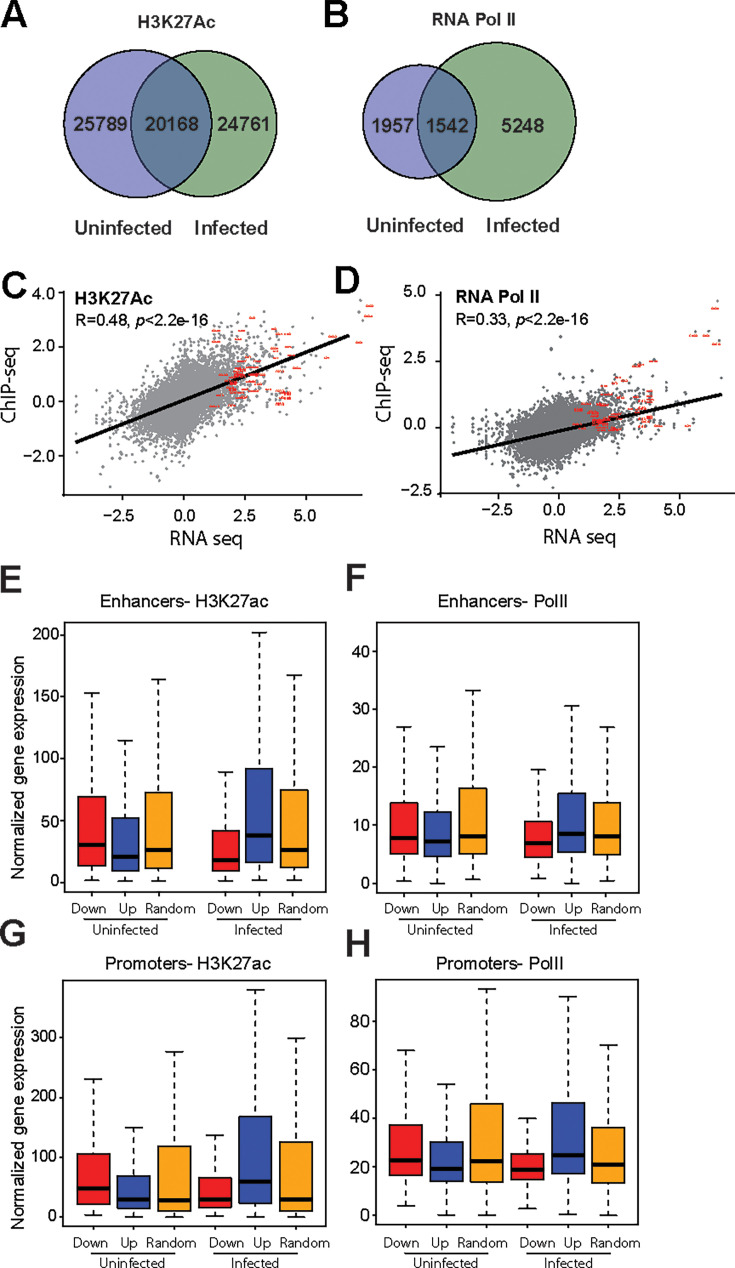
Altered H3K27Ac status promotes gene expression in infected cell population. (**A and B**) Venn diagram showing the number of sites enriched for H3K27Ac and Pol II upon *M. bovis* BCG infection of THP-1 MDMs. (**C and D**) Scatter plot showing the correlation in fold change values between gene expression from RNA-seq and H3K27Ac ChIP-seq (**C**) or Pol II ChIP-seq (**D**) from uninfected and infected conditions. Genes labeled in red highlight a set of secreted cytokines that show correlated changes in promoter occupancy and gene expression. (**E–H**) Boxplots comparing the ChIP signal at the enhancers (non-promoter H3K27Ac peaks closest to genes) or promoters of all upregulated and downregulated genes by 1.5-fold change (0.05 *P*adj) or an equivalent number of random genes upon mycobacteria infection. (**A**) H3K27Ac signals on enhancers. (**B**) Pol II signals on enhancers. (**C**) H3K27Ac signals on promoters. (**D**) Pol II signals on promoters. *P*-values were calculated by the Wilcoxon two-sided rank-sum test.

To identify the genomic regions where H3K27Ac and Pol II loading are altered, we performed genome-wide chromatin immunoprecipitation (ChIP-seq) with H3K27Ac and Pol II antibodies in the THP-1 MDM population infected with *M. bovis* BCG expressing GFP. We observed significant genome-wide changes in both H3K27Ac and Pol II loading, with several sites gaining H3K27Ac as well as Pol II upon infection (Fig. 3A and B). In order to understand if hyper-acetylation of specific regions in the genome leads to altered transcription, we plotted the correlation of fold change values of the differentially expressed genes from RNA-seq under identical conditions with the H3K27Ac or Pol II signal at the nearest promoter and enhancer. The results show a significant positive correlation (Fig. 3C and D), confirming that the open chromatin state of the infected cells is causal for the alterations in the gene expression profiles. Interestingly, p300, the specific writer for H3K27Ac, does not change upon infection (see Fig. S4A at https://doi.org/10.6084/m9.figshare.32879825) with *M. bovis* BCG as well as *Mtb*, while other histone acetyltransferase genes, including KAT8, were upregulated, while specific histone deacetylases (HDACs) are downregulated early in infection (Fig. S4B). KAT8 is a well-characterized histone acetyltransferase that promotes chromatin accessibility and transcriptional activation (36, 37). We validated the infection-induced increase in KAT8 expression by RT-quantitative PCR (qPCR). We observed a significant upregulation of KAT8 following *Mtb* infection (see Fig. S4C and S6D at https://doi.org/10.6084/m9.figshare.32879825), consistent with recent work (37) showing robust induction of KAT8 during *Mtb* infection. In addition, acetyl-CoA synthetase (AceCS1) levels increase (Fig. S4D) during *Mtb* infection, suggesting combinatorial mechanisms in play to enhance H3K27Ac during *Mtb* infection. Together, these results suggest that infection-induced chromatin remodeling involves the induction of distinct acetyltransferases that may facilitate the widespread gain of H3K27Ac observed in infected macrophages.

To explore the relationship between epigenetic alterations and gene expression changes further, we plotted the strength of H3K27Ac at promoter proximal and distal sites for the up- and downregulated genes. As expected, we observed a significant gain in H3K27Ac and Pol II signals in genes that are upregulated, and loss of the signal in genes that are downregulated (Fig. 3E through H). Overall, these results show that host macrophage chromatin is altered by mycobacterium infection to adopt a more active and open configuration, accompanied by changes in Pol II occupancy and altered transcription.

**Fig 4:**
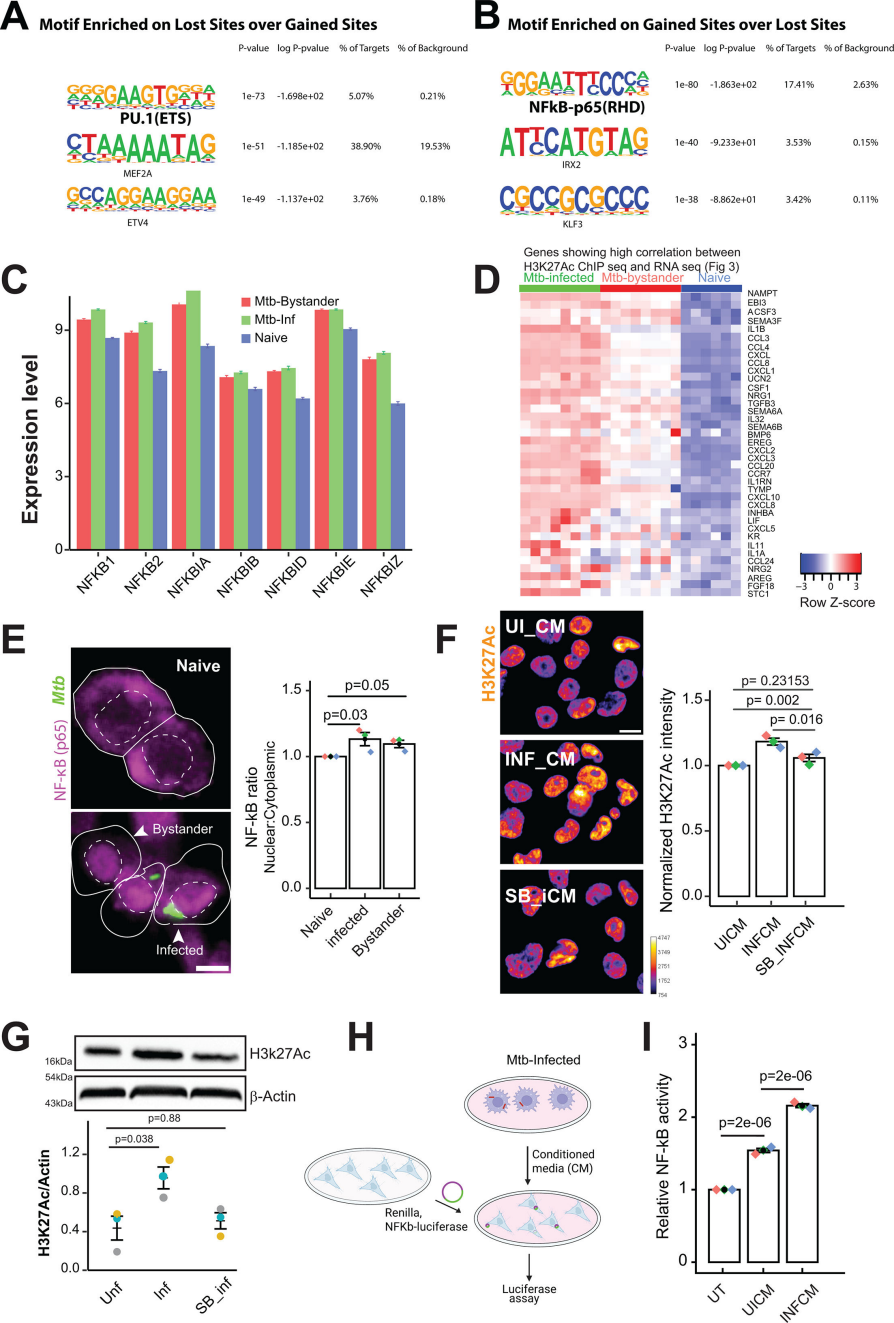
NF-κB-dependent secretory factors are major drivers for epigenetic alterations in the *Mtb*-bystander cells. (**A** and **B**) Motifs identified using differential motif analysis (HOMER) on the H3K27Ac sites lost (**A**) or gained (**B**) during *M. bovis* BCG infection in THP-1 MDMs. (**C**) Normalized mRNA expression levels of NF-κB-associated genes, which are upregulated in *Mtb*-bystander and *Mtb*-infected cells, compared to naive THP-1 MDMs. (**D**) Heatmap of selected differentially expressed genes that show correlated changes with H3K27Ac binding (Fig. 3C and D) between the *Mtb*-infected and *Mtb*-bystander subpopulations. Data set from Jani et al. (34) is used for panels **C and D**. (**E**) THP1 MDMs were infected with *Mtb*-mCherry for 48 h, fixed, and immunostained for NF-κB p65 subunit. Cells are segmented into infected and bystander subpopulations based on the presence of *Mtb*. Each dot represents an independent biological replicate. Data are mean ± SEM (*n* = 3). Scale bar in panel **E** is 10 μm. Data set from Jani et al. (34) is used for panels **C and D**. (**F**) THP-1 MDMs were treated with conditioned media from *Mtb*-infected, SB216763-treated infected, and uninfected cells. After 24 h, cells were fixed and immunostained for H3K27Ac. Representative images and quantification of normalized nuclear H3K27Ac intensity are shown. Each dot represents an independent biological replicate. Data are mean ± SEM (*n* = 3). Scale bar is 10 μm. (**G**) Western blot and quantification of H3K27Ac band intensity 48 h after *Mtb* infection in the presence or absence of SB-216763 in THP-1 MDMs; beta-actin was used as a loading control. Individual biological replicates are color-coded in the dot plot. Statistical significance was assessed using one-way ANOVA with Tukey’s multiple comparisons test. (**H and I**) HeLa cells were co-transfected with an NF-κB-responsive luciferase reporter and a *Renilla* luciferase construct for normalization, then treated with conditioned media from infected THP-1 MDMs. Luciferase activity was measured using a dual-luciferase assay, and NF-κB activity is expressed as relative luminescence units or fold change, normalized to *Renilla* and untreated controls. Each dot represents an independent biological replicate. Data are mean ± SEM (*n* = 3). Statistical significance was determined by one-way ANOVA followed by Tukey’s multiple comparisons test.

Since enhancer functions are directly linked with transcription factor binding, we performed motif analysis on the differential H3K27Ac sites. We observed that the PU.1 motif is significantly enriched on sites that are lost upon infection (Fig. 4A). PU.1 acts as a pioneering transcription factor (38), initiating the open chromatin state at enhancers. We hypothesized that these enhancers lose PU.1 binding upon infection, leading to the loss of H3K27Ac. To test this, we used PU.1 ChIP-seq data in THP1 cells (38) and compared it to the uninfected condition to assess if the lost sites bound with PU.1. Indeed, PU.1 occupancy mapped predominantly to the lost sites compared to gained sites or randomly chosen sites in the genome (see Fig. S5A at https://doi.org/10.6084/m9.figshare.32879825). These data identify PU.1 as an important mediator of mycobacteria-induced chromatin and epigenetic alterations, leading to loss of transcriptional program. Motif enrichment analysis in the genomic regions that gain H3K27Ac upon mycobacterial infection showed a significant enrichment for nuclear factor-κB (NF-κB) binding (Fig. 4B). Together, these results show that the overall open and active state of the chromatin upon mycobacterial infection is a net effect of specific loss and gained marks throughout the genome and modulated by factors such as PU.1 and NF-κB.

**Fig 5:**
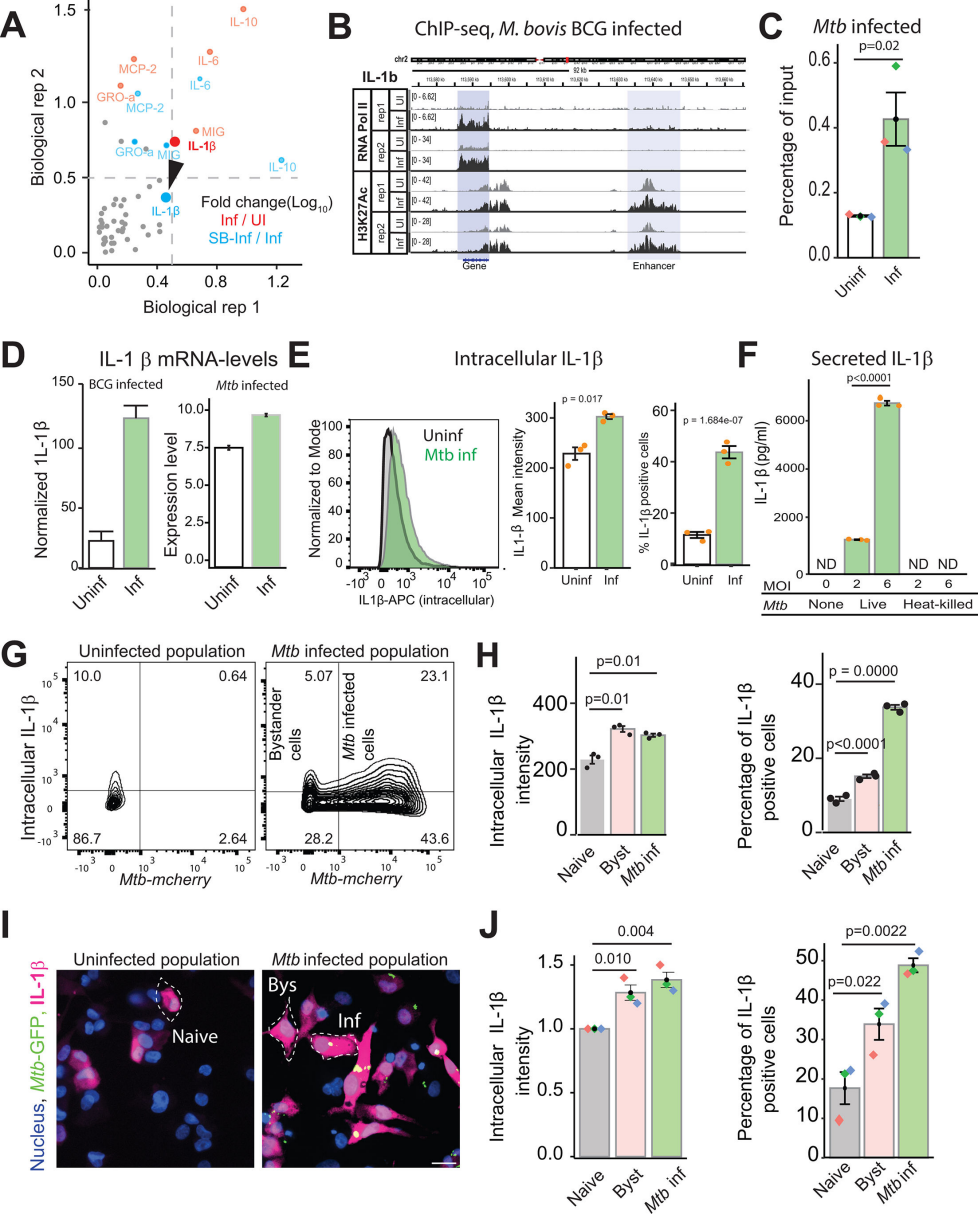
Secreted factors transmit epigenetic signals. (**A**) THP-1 MDMs were left unstimulated or infected with *Mtb* at 2 MOI for 48 h with or without SB-216763, a blocker of cytokine secretion. The log_10_ fold change in cytokine expression in the supernatant of infected over uninfected (red) and SB-216763 treatment over infection (blue) is shown as a scatter plot between two independent biological replicates. Dotted lines denote a threshold of 0.5-fold change. Cytokines showing substantial reduction upon SB-216763 treatment are highlighted. (**B**) Interactive Genomics Viewer browser screenshots for IL-1β genomic region showing the gain of H3K27Ac and RNA Pol II at the promoter and enhancer regions. (**C**) ChIP-qPCR analysis of H3K27Ac enrichment at the IL-1β promoter in THP-1 MDMs under uninfected and *Mtb*-infected conditions. Data are presented as a percentage of input. Each dot represents an independent biological replicate; statistical significance was determined using Student’s *t*-test; error bars indicate mean ± SEM. (**D**) Normalized read counts for the IL-1β gene from the RNA-seq data upon *M bovis* BCG infection, and normalized mRNA expression levels of the IL-1β gene upon *Mtb* infection. (**E**) Flow cytometric analysis of intracellular IL-1β staining from THP-1 MDMs infected with *Mtb*-mCherry, performed independently three times. Bar graphs show the mean fluorescence intensity (MFI) of intracellular IL-1β (middle) and the percentage of IL-1β-positive cells (right) in *Mtb*-infected and uninfected cells. A total of 30,000 events were collected per condition; dots denote the technical replicates. (**F**) IL-1β amounts in the supernatant of THP1 monocyte derived macrophages infected with live or heat-killed Mtb at the indicated MOI measured by ELISA. (**G and H**) Contour plot showing intracellular IL-1β in infected, bystander, and uninfected cells. MFI of IL-1β and percentage of cells positive for IL-1β are shown in the bar plot. (**I and J**) Representative images showing intracellular IL-1β staining in naive, bystander, and infected THP-1 MDMs. Quantification of IL-1β fluorescence intensity and the percentage of IL-1β-positive cells are shown. Data are presented as mean ± SEM of three biological replicates. Scale bar is 25 μm. For panels** F**,** H, **and **J**, statistical significance was assessed using one-way ANOVA with Tukey’s multiple comparisons test. Dashed lines in panel **I** denote the cell boundary.

The specific enrichment of NF-κB on the H3K27Ac-gained sites in infected cells prompted us to assess NF-κB-dependent genes in our RNA-seq data. Indeed, several NF-κB-dependent cytokines are among the top correlated genes between our ChIP-seq and RNA-seq data sets (Fig. 3C and D), many of which also show enhanced secretion into the supernatant upon mycobacterial infection (Fig. 5A). Since the ChIP-seq and RNA-seq were performed in *M. bovis* BCG-infected cells, we tested if these results are comparable in *Mtb* infection. Interestingly, the expression levels of several NF-κB genes show substantial similarity between *Mtb-*infected and *Mtb*-bystander cells (Fig. 4C), while being distinct from naive uninfected cells, suggesting upregulation of NF-κB itself during *Mtb* infection. We further analyzed the expression of NF-κB-dependent genes that were correlated in our RNA-seq and ChIP-seq data set from *M. bovis* BCG-infected cells (Fig. 4D) in the *Mtb*-bystander cells. Heatmap analysis reveals that the expression of NF-κB-dependent genes of the *Mtb*-bystander subpopulation is substantially similar to that of the infected cell subpopulation and distinct from that of the naive cells (Fig. 4D). These results show that in both *M. bovis* BCG and *Mtb* infections, NF-κB-dependent genes show a coordinated response in infected as well as bystander cells. To experimentally verify these findings, we stained *Mtb-*infected cells with anti-NF-κB p65 antibody and assessed the nuclear translocation of NF-κB between *Mtb*-infected and *Mtb*-bystander cells. The results (Fig. 4E) show that while NF-κB was largely cytosolic in naive cells, nuclear translocation was seen in *Mtb*-infected cells, as reported earlier (39), and importantly in *Mtb*-bystander cells. Together, these results suggest that the epigenetic and gene expression alterations induced by *Mtb* in the infected cells could be transmitted to the bystander cells in an NF-κB-dependent manner to alter gene expression in both infected and bystander subpopulations.

We next sought to identify the mechanisms by which information is transmitted from infected to bystander cells to alter epigenetic changes in bystander cells and render them distinct from the naive cells. Our definition here of bystander cells as cells in the same population of *Mtb-*infected cells but not harboring visible *Mtb* does not rule out the possibility that the bystander cells are under direct influence from the bacteria. For example, the cell having been previously infected, and the bacteria either cleared or escaped from the cell without killing it (40). Any alterations in such cells could be due to direct contact with *Mtb* and not the true bystander effect. To rule out such a scenario in the epigenetic alterations in bystander cells, we added media from *Mtb*-infected cells (*Mtb-*conditioned media) to naive cells and assessed H3K27Ac levels. The results (Fig. 4F) show that treatment with *Mtb-*conditioned media was indeed sufficient to enhance H3K27Ac levels in naive cells, suggesting a role for secretory factors in modulating epigenetic changes in bystander cells. Given the secretory nature of several cytokines, their dependence on NF-κB and their emerging roles in epigenetic regulation (41–43), we hypothesized that cytokines carry information of infection to neighboring cells to propagate epigenetic modifications. In support of this, conditioned media (CM) obtained from *Mtb*-infected cells treated with SB216763, a known blocker of cytokine secretion (44–47), abrogated the increase in H3K27Ac as assessed by imaging (Fig. 4F) or immunoblotting (Fig. 4G). Furthermore, non-immune cells show a similarly enhanced H3K27Ac status upon treatment with media conditioned by *Mtb*-infected THP-1 MDM cells (see Fig. S5B at https://doi.org/10.6084/m9.figshare.32879825), suggesting conservation of NF-κB-dependent epigenetic modification across cell types. Finally, to experimentally validate whether the NF-κB motif is functionally active, cells were co-transfected with an NF-κB-responsive luciferase construct and a *Renilla* luciferase control plasmid and exposed to conditioned media from *Mtb*-infected macrophages. The results showed enhanced NF-κB-dependent luciferase activity in conditioned media-treated cells (Fig. 4H and I), confirming that the enriched NF-κB motifs are transcriptionally active and mediate the bystander effect. These results confirm that the identified NF-κB motif is functionally active and the associated epigenetic modifications translate into corresponding changes in gene expression. Together, these results identify that the NF-κB motif is activated in mycobacteria-infected as well as conditioned media-treated cells and functionally links infection-induced cytokine signaling with open chromatin states, enhancer activation, and transcriptional reprogramming.

### Secreted factors transmit epigenetic signals

We next sought to identify if specific cytokines could mediate the underlying epigenetic alterations in *Mtb*-infected and bystander cells. We reasoned that such a molecule should (i) be under mycobacterium-mediated epigenetic control, (ii) be a secretory cytokine detectable in the supernatant, (iii) be under NF-κB regulation, and (iv) cause epigenetic alteration on its own in naive macrophages. To identify such a mediator, we profiled the cytokines released in the medium upon *Mtb* infection in the presence or absence of SB216763 using a cytokine array. Results from two independent biological replicates show several cytokines induced during *Mtb* infection and specifically decreased upon SB216763 treatment (Fig. 5A), with IL-1β showing a substantial and reproducible change. Closer examination of the ChIP-seq data set for the IL-1β gene region from *M. bovis* BCG-infected THP-1 MDMs showed an increase in H3K27Ac binding both at its promoter and enhancer regions, showing that the IL-1β locus is directly regulated by *M. bovis* BCG infection. To check if this is also the case during *Mtb* infection, we performed H3K27Ac ChIP-qPCR with primers targeting the IL-1β promoter. This analysis showed significant enrichment of H3K27Ac at the IL-1β promoter following *Mtb* infection (Fig. 5C), demonstrating epigenetic activation of IL-1β as a conserved response during both *M. bovis* BCG and *Mtb* infections. Furthermore, enhanced Pol II binding was observed on the promoter and gene body of IL-1β (Fig. 5B), indicating active transcription of the gene. Correspondingly, IL-1β mRNA read counts were significantly higher in mycobacterium-infected cells (Fig. 5D). Intracellular staining for IL-1β showed an increase upon *Mtb* infection, as detected by both imaging and flow cytometry (Fig. 5E, I, and J), showing enhanced translation. Furthermore, enhanced secretion of IL-1β was detected in the supernatants from live but not heat-killed *Mtb*-infected cells (Fig. 5F) in a dose-dependent manner, similar to other reports (48). These results show a distinct hierarchy in the modulation of IL-1β secretion upon both *M. bovis* BCG and *Mtb* infections, with epigenetic alterations in the IL-1β gene region, resulting in enhanced transcription, translation, and secretion of IL-1β. Our previous data (Fig. 4) showed enhanced NF-κB nuclear activation and activity in bystander cells. We next checked whether IL-1β expression can be directly detected in *Mtb*-bystander cells by assessing intracellular IL-1β in the infected and bystander subpopulations by flow cytometry as well as imaging. The results (Fig. 5G through J) show a significant increase in both the percentage of cells producing IL-1β as well as intracellular IL-1β intensity compared to naive cells, confirming that indeed *Mtb*-bystander cells produce IL-1β. Together, these findings identify IL-1β as a key cytokine that is induced not only in infected macrophages but also in *Mtb*-bystander cells, supporting a role for paracrine IL-1β signaling in propagating infection-induced responses across the macrophage population.

### IL-1 signaling is important for bystander remodeling

**Fig 6:**
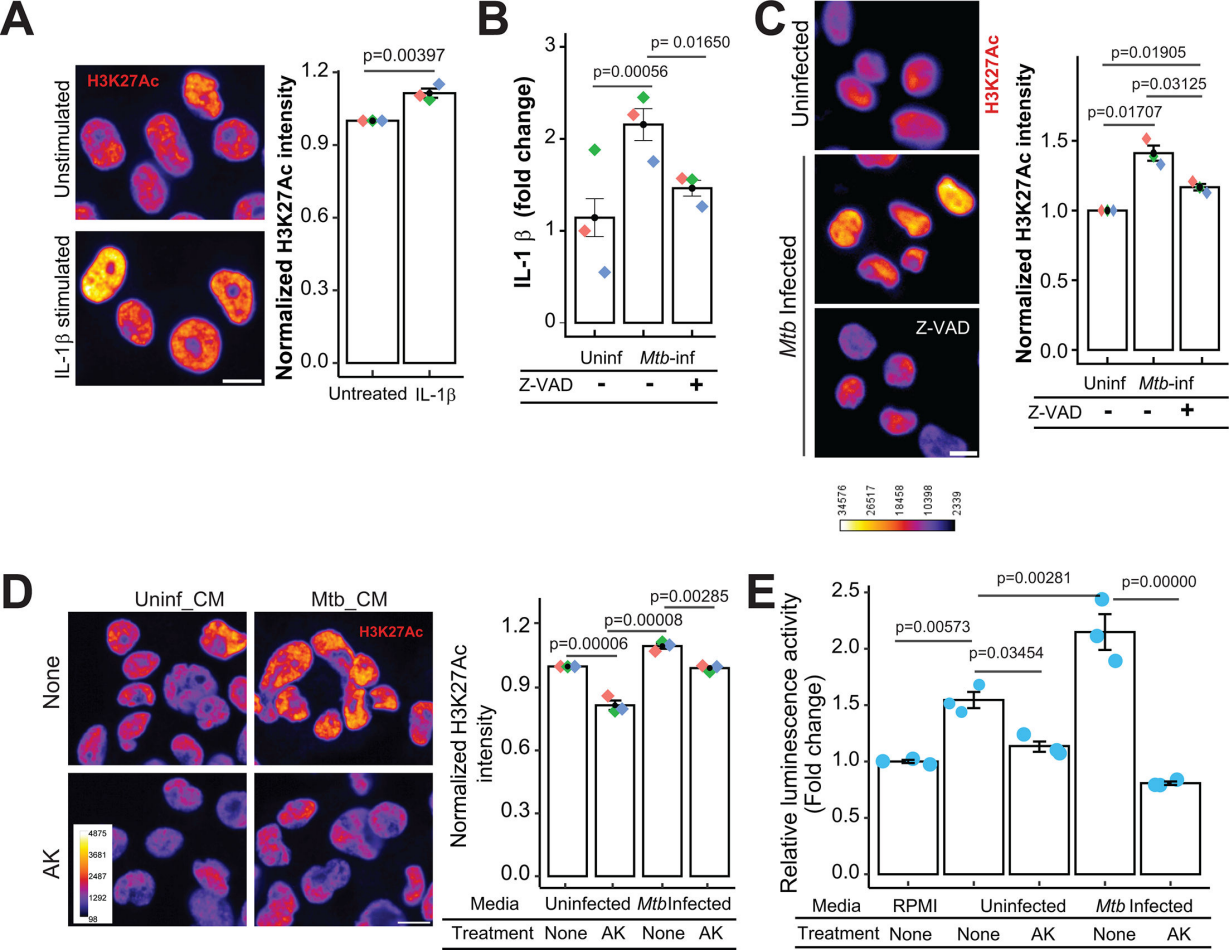
IL-1 signaling is important for bystander cell reprogramming. (**A**) Representative immunostaining of H3K27Ac of naive THP-1 MDMs stimulated with IL-1β. Each dot represents an independent biological replicate; statistical significance was determined using Student’s *t*-test; error bars indicate mean ± SEM. (**B**) IL-1β levels in the supernatant of THP-1 MDMs upon *Mtb* infection with or without Z-VAD. Each dot represents an independent biological replicate. Data are mean ± SEM (*n* = 3). (**C**) THP-1 MDMs were infected for 48 h in the presence or absence of Z-VAD, followed by fixation and immunostaining with H3K27Ac antibody. Representative images and quantification of normalized nuclear H3K27Ac intensity are shown. Each dot represents an independent biological replicate; data are shown as mean ± SEM. Scale bar is 10 μm. (**D**) THP-1 MDMs were either pretreated with anakinra (recombinant human interleukin-1Ra) for 24 h or left untreated, then subjected to *Mtb*-CM treatment, and immunostained with H3K27Ac antibody. Each dot represents an independent biological replicate; data are shown as mean ± SEM. Scale bar is 10 μm. (**E**) HeLa cells were co-transfected with an NF-κB-responsive luciferase reporter plasmid along with a *Renilla* luciferase construct for normalization. Following transfection, cells were pretreated with anakinra for 30 min and then treated with conditioned media as indicated. Luciferase activity was measured using a dual-luciferase assay system, and NF-κB activity is presented as relative luminescence units or fold change normalized to *Renilla* and relative to untreated controls. Data represent mean ± SEM of three technical replicates from a representative biological replicate. Experiments were performed in two independent biological replicates. For panels **B**, **C**, **D,** and** E**, statistical significance was assessed using one-way ANOVA with Tukey’s multiple comparisons test.

To test if IL-1β could directly modulate the epigenetic changes observed in bystander cells, we stimulated naive macrophages with recombinant IL-1β and assessed changes in H3K27Ac levels by quantitative imaging. The results (Fig. 6A) show a significant increase in nuclear H3K27Ac. In line with previous reports (49), these results show that IL-1β on its own causes epigenetic remodeling, resulting in gene expression alterations. Our earlier experiments using SB-216763 suggested that cytokine secretion contributes to H3K27Ac enrichment; however, SB-216763 is a broad inhibitor of cytokine release. We therefore sought to directly test the specific contribution of IL-1β secretion to epigenetic modification during *Mtb* infection. To this end, we treated *Mtb*-infected macrophages with the pan-caspase inhibitor Z-VAD, which blocks inflammasome-dependent IL-1β maturation and secretion (50, 51). Z-VAD treatment significantly reduced infection-induced IL-1β secretion and, notably, attenuated the enhanced nuclear H3K27Ac (Fig. 6B and C), demonstrating that IL-1β secretion contributes to chromatin remodeling during infection. We next asked whether IL-1β signaling in recipient bystander cells is required for the epigenetic changes induced by *Mtb*-conditioned media. THP-1 MDMs were pre-treated with anakinra, a well-established IL-1 receptor antagonist (41, 52), prior to exposure to *Mtb*-conditioned media. Anakinra pre-treatment markedly reduced nuclear H3K27Ac levels compared to cells treated with conditioned media alone (Fig. 6D), demonstrating a crucial role for IL-1β signaling in modulating epigenetic alterations in *Mtb*-bystander cells. Notably, under both inhibitory conditions, H3K27Ac levels did not return to baseline. Further conditioned media from uninfected cells also led to a reduction in H3K27Ac during anakinra pre-treatment. Together, these data suggest that additional infection-induced signals contribute to epigenetic modification in bystander cells.

To determine whether IL-1-dependent paracrine signaling activates the NF-κB motif in bystander cells, we performed an NF-κB luciferase reporter assay. Similar to previous results (Fig. 4I), *Mtb*-conditioned media induced robust NF-κB-dependent luciferase activity, although basal activation was observed in media from uninfected cells. Importantly, IL-1 receptor blockade significantly reduced the reporter activity, indicating that this paracrine NF-κB activation in bystander cells is IL-1-dependent (Fig. 6E).

Next, we examined the functional consequences of IL-1 signaling during *Mtb* infection. Blocking IL-1 receptor signaling with anakinra increased infection frequency and bacterial burden (see Fig. S6A at https://doi.org/10.6084/m9.figshare.32879825), consistent with a role for IL-1 signaling in restricting mycobacterial growth (41). Inhibition of IL-1 signaling by anakinra but not Z-VAD had a partial effect on the bystander restriction phenotype during second Mtb infection (Fig. S6B and C). Collectively, these results identify IL-1 signaling as an important contributor to the propagation of infection-induced epigenetic remodeling from infected macrophages to surrounding bystander cells.

### Bystander cells exhibit features of trained immunity

**Fig 7:**
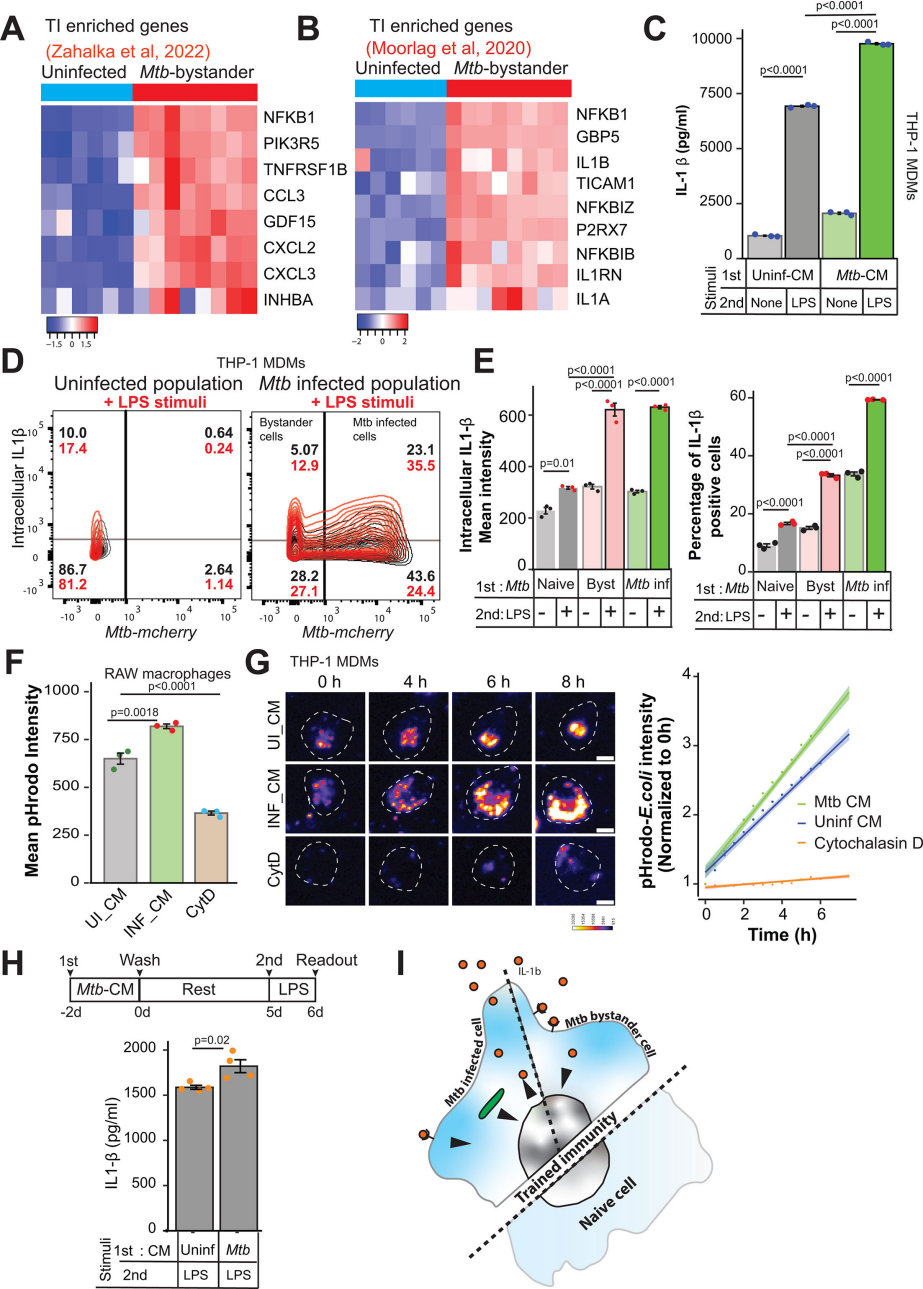
Coordinated epigenetic modifications in the infected and bystander subpopulations promote trained immunity in the population. (**A and B**) Heatmap showing the expression profiles of selected TI-enriched genes from two different published TI regimes in the RNA-seq data from the *Mtb*-infected and *Mtb*-bystander subpopulations. (**C**) THP-1 MDMs were treated with *Mtb*-CM for 24 h and re-stimulated with 10 ng/mL LPS for 24 h. IL-1β in the supernatant was measured by enzyme-linked immunosorbent assay (ELISA). Results are compiled from three technical replicates and are representative of at least three biological replicates. Statistical significance was determined with one-way ANOVA using Tukey’s multiple comparisons test; error bars indicate mean ± SEM. (**D**) Representative contour plot of intracellular IL-1β expression of unstimulated *Mtb-*infected THP-1 MDMs (gray) compared with the LPS-stimulated *Mtb*-infected macrophages (red). The quadrant gates were set using corresponding unstained controls. Values represent the proportion of cells within the indicated gates, without (black) or with (red) LPS stimulus. (**E**) Mean fluorescence intensity of intracellular IL-1β (left) and the percentage of IL-1β-positive cells (right) between the different conditions. A total of 30,000 events were collected per condition; dots denote the mean of technical replicates. Statistical significance was determined with one-way ANOVA using Tukey’s multiple comparisons test; error bars indicate mean ± SEM. (**F**) Flow cytometry analysis of CM-treated RAW 264.7 macrophages pulsed with pHrodo *E. coli*. Cytochalasin D was used as an assay control. Significance was assessed by one-way ANOVA using Tukey’s multiple comparisons test; error bars indicate mean ± SEM. (**G**) Time-lapse images of THP-1 MDM pulsed with pHrodo *E. coli*. The right panel shows the fluorescence intensity of intracellular pHrodo-*E. coli* in the same cell over the indicated time period normalized to the initial time point of that cell. Data are from at least 30 cells per condition and are representative of three independent biological replicates. Data point represents the mean fluorescence intensity of pHrodo-*E. coli* from all the cells with standard error in the shaded region. Significance was assessed by one-way ANOVA using Tukey’s multiple comparisons test. (**H**) A schematic of the training protocol in THP-1 MDMs. Cells were trained with *Mtb* CM for 24 h and re-stimulated with LPS following a 5-day rest period. IL-1β production in the supernatant was measured by ELISA as a response to secondary stimuli. *P*-values are obtained using Student’s *t*-test; the data shown are the mean  ±  SEM and are representative of three independent biological replicates. (**I**) Model summarizing the key results of the study.

We next aimed to determine whether the growth-restrictive effect observed in bystander cells upon reinfection was specific for *Mtb* or reflected a broader antimicrobial immune state. Trained immunity, or memory of innate immune cells, is emerging as an important paradigm in immunology, where innate immune cells trained with a primary stimulus respond better to a subsequent stimulus compared to untrained cells. Epigenetic modifications are well established as drivers of TI response (53), while *Mycobacterium* infections are commonly used as primary stimuli in experimental TI regimens (28, 29, 54, 55). Given these parallels with our observations, we hypothesized that the propagation of epigenetic alterations from infected to bystander cell subpopulations could promote broader training in the infected cell population. To test this, we compared the gene expression signatures of *Mtb*-bystander cells (34) with TI signature genes obtained from two different TI experimental systems, namely, mouse alveolar macrophages exposed to *Streptococcus pneumoniae* (6 days *in vivo* lipopolysaccharide [LPS] training) or human monocytes stimulated with β-glucan (24 h of stimulation followed by 5 days of rest) (41, 56). In both cases, despite the differences in species, nature, and duration of the stimuli, we observed a strong overlap of the TI gene signatures with *Mtb*-bystander but not naive cells (Fig. 7A and B). These results further reinforce the distinction between naive and bystander cells, show the robustness of the bystander response, and suggest that bystander cells acquire a trained state that could enhance their response to subsequent microbial encounters. To test this experimentally, we mimicked the bystander condition by treating naive THP-1 MDMs with *Mtb*-conditioned media, then challenged them with lipopolysaccharide and measured IL-1β production as a readout of responsiveness (57). As expected, cells exposed to both uninfected and *Mtb*-conditioned media exhibited enhanced IL-1β production in response to the secondary stimulus (Fig. 7C). Importantly, cells treated with *Mtb*-conditioned media showed a significantly greater response, suggesting that exposure to *Mtb* infection-derived factors is sufficient to induce a trained immunity-like state. To check if similar alterations can be observed directly in the bystander cells in an *Mtb*-infected population, we stimulated the *Mtb*-infected population with LPS and stained for intracellular IL-1β, which allowed assessment of alterations in IL-1β production at a single-cell level. The results show that both *Mtb*-infected and bystander cells respond better to LPS compared to naive cells (Fig. 7D and E). To further substantiate this result, we used another standard readout of TI, namely phagosome maturation. We pulsed macrophages treated with conditioned media from either uninfected or *Mtb*-infected cells with *Escherichia coli* tagged to pHrodo, a pH-sensitive dye, and assessed the intracellular fluorescence of *E. coli* by flow cytometry (Fig. 7F) or imaging (Fig. 7G). Higher fluorescence indicates delivery of *E. coli* to the acidic lysosomes. Cytochalasin D, a blocker of phagocytic uptake, was used as an assay control. The results show a significant increase in intracellular pHrodo-*E. coli* fluorescence in cells treated with *Mtb*-conditioned media compared to control cells, showing an enhancement in phagosome maturation. These results provide functional evidence that bystander macrophages acquire a TI-like state during *Mtb* infection. TI response is typically linked to a “rest” period, during which the primary stimulus is removed. To assess whether this applies to *Mtb*-bystander cells, we implemented a 5-day rest period (Fig. 7H) after exposure to *Mtb*-conditioned media. While IL-1β levels were undetectable at the end of the rest period in conditioned media from both uninfected and *Mtb*-infected cells, *Mtb*-conditioned media exhibited a significantly enhanced production of IL-1β in response to secondary LPS stimulation (Fig. 7H). Collectively, these results demonstrate that *Mtb*-bystander cells exhibit properties of trained immunity for better control of subsequent infections.

## DISCUSSION

Non-infected cells in an infected population, called bystander cells, have typically been assigned a passive role in infection outcomes. Emerging literature, however, shows that the gene expression signatures of bystander cells from *Mtb*-infected mice are distinct from naive cells (7, 8, 58). Our results here show that bystander cells acquire enhanced responsiveness to subsequent stimuli, including heightened control of a second round of *Mtb* infection. Bystander cells show gene expression patterns similar to the infected cells, mediated by epigenetic alterations driven by cytokines (predominantly IL-1β) secreted from infected cells. Interestingly, IL-1β secretion itself is a direct consequence of *Mtb* infection-driven epigenetic alteration in the infected cell, leading to NF-κB activation. Thus, epigenetically driven gene expression changes in infected cells influence surrounding bystander cells, resulting in heightened population-wide responsiveness resembling trained immunity.

Mycobacterial infection alters macrophage chromatin organization to favor a net genome-wide active chromatin state. Nevertheless, distinct sites in the genome, such as specific enhancers, are both gained and lost, underlining the specificity in the modulation. Our results further identify specific enhancer signatures that drive gene expression changes, most prominently the enrichment of NF-κB motif in gained regions and PU.1 in the lost regions (Fig. 4A and B). PU.1 is a pioneering transcription factor known for its role in immune cells and its ability to shape the chromatin landscape and modulate the expression of hundreds of genes, including other transcription factors, growth and differentiation factors, adhesion molecules, and signaling mediators (38, 59, 60). By selectively closing PU.1-interacting motifs, *Mtb* could influence a variety of distinct regulatory elements and genes they control. Interestingly, many PU.1-dependent genes, as well as PU.1 levels themselves, increase upon exposure to *Mtb* (61, 62), suggesting additional layers in the interaction.

The similarity of these results between *M. bovis* BCG and *Mtb* infections indicates conserved responses between the two for these epigenetic modifications. The requirement of live *Mtb* for the chromatin alterations supports earlier reports of specific *Mtb* factors promoting host histone modifications (17–20), as well as the need for continued mycobacterial presence for sustained chromatin alterations. Several mycobacterial products and host signaling pathways, such as TLRs, can activate NF-κB signaling during mycobacterial infection (63, 64). Our results suggest the possible contribution of a specific histone acetyltransferase, such as KAT8 (36), but not the canonical HAT p300. Increased expression of acetyl-CoA synthetase (AceCS1) suggests that metabolic rewiring may support the widespread H3K27Ac gain observed during infection. However, given the redundancy of HAT and HDAC networks and their connection with metabolic pathways (65), the full signaling cascade leading to chromatin remodeling during mycobacterial infections needs to be clearly defined.

In this work, we focus on the NF-κB binding motif, which gains H3K27Ac upon mycobacterial infection, to adopt an open configuration, resulting in the strong positive correlation between promoter accessibility for H3K27Ac and gene expression for several NF-κB-dependent cytokines. These cytokines released from infected cells induced epigenetic alterations in the recipient cells, including bystander cells, resulting in gene expression changes resembling the infected cells. NF-κB reporter assays demonstrated functional activation of NF-κB-responsive regulatory elements, providing a mechanistic link between infection-induced epigenetic changes and gene expression in both infected and bystander cells. A crucial outcome of this alteration is the enhanced transcription of specific cytokine genes, as exemplified in our work by IL-1β.

IL-1β production in the cell is tightly regulated at multiple levels, including transcription, post-translational processing, and non-canonical secretion (66–68). Our results here show a concerted mechanism for enhanced IL-1β secretion upon mycobacterial infection across these layers of regulation and support a functional link between infection-induced epigenetic remodeling and enhanced IL-1β secretion. Importantly, secreted IL-1β contributes to further epigenetic changes in bystander cells. In support of this, inhibiting IL-1β signaling either by blocking secretion or by receptor engagement in the recipient cells reduced the epigenetic modification in both infected and bystander cells. Collectively, these data favor a model in which specific cytokines, predominantly IL-1β, secreted into the medium by the *Mtb*-infected cell, act on bystander cells, altering the epigenome to promote an open chromatin conformation. Perturbation of IL-1β signaling did not completely abolish epigenetic alterations in bystander cells, suggesting that additional mediators likely contribute to this intercellular communication. Furthermore, unlike IL-1β, bystander cells do not produce TNF-α (69), another NF-κB-dependent cytokine (70), suggesting distinct regulatory mechanisms that could define the specificity of the epigenetic alterations and gene expression in bystander cells. Importantly, these bystander alterations are not limited to macrophages but are also observed in epithelial cells. This could possibly explain the altered epigenome and gene expression patterns seen in diverse distal cells such as circulating lymphocytes in TB patients (6), HSC in bone marrow (29), or PBMC from BCG-vaccinated humans (42). Our results using intracellular cytokine labeling show IL-1β production in the bystander cells, suggesting a positive feedback loop, which could help in such distal propagation of epigenetic signatures from *Mtb*-infected cells. Likely, such a mechanism is tightly regulated, since uncontrolled propagation could promote systemic hyperinflammation. IL1RN, the antagonist inhibiting the activity of IL-1β (71), is also expressed in bystander cells together with IL-1α and IL-1β, suggesting possible mechanisms of quenching the IL-1β signaling cascade initiated by mycobacterial infection. IL-1β is well known to promote pyroptotic cell death during *Mtb* infection *in vitro* (31, 72); however, whether IL-1β-driven pyroptosis contributes to cell death *in vivo* remains largely unexplored and represents an important gap in the field. The observed correlation of secretory IL-1β levels with *Mtb* clinical strains (73, 74), disease severity (75, 76), and transmission (77) warrants investigations into potential differences in the epigenetic alterations under these conditions.

What could be the role of epigenetic alterations in bystander cells? We reasoned that epigenetic alterations in the bystander cells could prepare the cells to respond better to a subsequent round of infection. Trained immunity is an emerging paradigm in immunology that attributes innate immune cells the property of “memory,” loosely defined as providing greater protection against reinfection (54). Epigenetic alterations are causal in promoting and sustaining trained immunity, as effector immune/defense pathway genes remain poised in trained cells for rapid action for a subsequent heterologous stimulus (28, 78). Here, we show significant conservation in the gene expression signatures of *Mtb*-bystander cells with macrophages from two different training regimes (41, 56). These results suggest that *Mtb*-bystander macrophages could be undergoing training. We experimentally tested this by subjecting *Mtb*-conditioned media-treated cells or *Mtb*-bystander cells to a commonly used secondary stimulus, LPS (56), and assessed training using well-established readouts such as phagosome maturation and cytokine secretion (53). Our results demonstrate that *Mtb*-bystander macrophages indeed exhibit an elevated response to the secondary stimuli, thereby showing properties of trained macrophages.

Conventional training paradigms typically report the outcomes for the entire cellular population (29, 42), but not specific cellular subpopulations, such as bystander cells. Our results here demonstrate the flow of information from a subset of cells directly receiving the primary stimuli (mycobacterial infection) to the bystander cells through soluble cytokine mediators and their consolidation in the bystander cells as epigenetic imprints. Importantly, our results show that IL-1β itself enhances the H3K27Ac in recipient cells, in agreement with previous findings of a key role for IL-1β in trained innate immunity (41, 52, 79). The mycobacterial triggers initiating the epigenetic reprogramming in infected cells need to be clearly determined. It will be important to determine if and how the individual *Mtb* factors identified to induce epigenetic alterations (17–22) act in concert to determine the selective opening of chromatin in the genome to promote training in the infected cell population. Conventional experimental TI regimes typically involve a “rest” period (54, 80) where the primary stimulus is withdrawn. However, “living” stimuli such as *M. bovis* BCG or *Mtb* could provide continuing inputs from within the cell due to their sustained intracellular residence, resulting in differences from other training regimes. Indeed, different stimuli such as LPS, β-glucan, or BCG led to the induction of distinct TI programs (54). Overall, our results support a model in which IL-1β-dependent intercellular signaling contributes to the transfer of infection-induced epigenetic reprogramming from infected to bystander cells, shaping the induction as well as propagation of trained immunity in the population.

An external stimulus, particularly infection with bacteria, typically directly affects only a subpopulation of the cells (81, 82), resulting in significant cellular heterogeneity. Even within an infected population, multiple outcomes occur at a single-cell level (40, 81). How the intracellular nature of mycobacteria and the heterogeneous host cell response at a single-cell level impact the kind and quality of TI programs is not known. Our results suggest cytokine-driven epigenetic reprogramming in the entire population as a major equilibrating factor that could counter the effect of single-cell variations in the infection outcome. In support of this, our single-cell analysis shows an equilibration of the nuclear H3K27Ac levels in the entire cellular population, irrespective of the *Mtb* load in an individual cell, but dependent on the population-level MOI used for infection. The heterogeneity of cellular responses for primary and secondary stimuli during training at a single-cell level is largely unstudied. Interestingly, our intracellular IL-1β staining shows that only a subset of both *Mtb-*infected and bystander macrophages produces IL-1β, underlining the heterogeneity of responses at a single-cell level, while also suggesting that a subset of responder cells has a dominant effect in generating population-wide response. Why only a subset of infected and bystander cells produce IL-1β is currently unclear; emerging single-cell multi-omics technologies could help uncover the relationship between open chromatin states and gene expression at a single-cell level and address whether they provide additional regulatory roles for the population. Identifying what determines the subset of such responders will be crucial to gain a deeper understanding of trained immunity and also in developing optimal strategies to modulate it for therapeutic benefits. Regulating trained immunity is shown as a powerful target in several diseases (54). Our identification of IL-1β as a crucial player in propagating TI to *Mtb*-bystander cells suggests modulating IL-1β as a possible strategy to influence the extent and level of signal propagation that could dampen or augment population-level trained immunity. Overall, we uncover a mechanism involving cytokine-mediated intercellular communication and epigenetic reprogramming that promotes trained immunity-like responses in bystander cells within a mycobacteria-infected population, leading to a heightened immune response in the entire cellular population.

## MATERIALS AND METHODS

### Cell culture and infection

THP1 monocytes were cultured in RPMI 1640 (GibcoTM 31800022) supplemented with 10% fetal bovine serum (GibcoTM 16000-044) and 0.01% antibiotic-antimycotic (GibcoTM 15240-062). THP1 monocytes were differentiated to macrophages by treatment with 20 ng/mL of phorbol-12-myristate-13-acetate (PMA, Sigma-Aldrich, P8139) for 16 h followed by incubation in PMA-free RPMI 1640 medium for 2 days and used for infections. THP1 monocyte-derived macrophages were incubated with mycobacteria for 4 h using 2 MOI, unless stated otherwise, followed by multiple washes with RPMI medium to remove extracellular bacteria and incubated with fresh RPMI medium for 48 h or stated otherwise. All infections with *Mycobacterium tuberculosis* were done in a BSL-III facility. Depending on the specific assay being conducted, cells were either fixed with 4% paraformaldehyde (PFA) for immunofluorescence analysis or lysed using radioimmunoprecipitation assay (RIPA) buffer for western blotting.

### *Mtb* culture

Mycobacterial cultures were grown in Middlebrook 7H9 broth (BD Biosciences, 271310) supplemented with Middlebrook OADC Enrichment (BD Biosciences, 211886), 5% glycerol (G5516, Sigma-Aldrich), and 0.05% Tween 80 (Sigma-Aldrich, P4780). Mycobacterial cultures were used at mid-log phase (OD_600_ = 0.6–0.8). All experiments involving *Mycobacterium tuberculosis* were conducted in a biosafety level III (BSL-III) facility. To maintain the plasmid constructs in *Mycobacterium tuberculosis*, kanamycin (Sigma-Aldrich, K1876) was used at a concentration of 25 µg/mL for *Mtb* mCherry H37Rv. *M. bovis* BCG constitutively expressing GFP was cultured in a BSL-II lab with kanamycin 25 µg/mL in 7H9 broth.

### Isolation of bone marrow-derived macrophages

For bone marrow-derived macrophage (BMDM) culture, bone marrow was flushed out from the tibia and femur using a syringe filled with RPMI medium. To produce differentiated BMDMs, bone marrow cells were plated in RPMI medium supplemented with 10% FBS and 20% L929 conditioned media. BMDMs were harvested and plated for infection assays after 7 days.

### Immunostaining, imaging, and image analysis

For immunostaining H3K27Ac, differentiated THP1 macrophages after infection were fixed with 4% paraformaldehyde, washed with 1× phosphate-buffered saline (PBS), and permeabilized with 0.2% Triton X-100 permeabilization buffer (Sigma-Aldrich T8787), 1% fetal bovine serum (GibcoTM 16000-044), 1% bovine serum albumin (Sigma-Aldrich, A2153) in PBS for 12 min at room temperature. Primary antibodies H3K27Ac (Abcam, ab4729), NF-κB p65 (CST, 6956S), and phospho-Rpb1 CTD (Ser2) rabbit (CST, 13499), phospho-Rpb1 CTD (Ser5) rabbit (CST, D9N51), and anti-F4/80 (Abcam, ab6640) were prepared in antibody dilution buffer (1% FBS and 1% BSA in 1× PBS) and incubated overnight at 4°C. After washing with PBS-T (0.001% Tween-20 in PBS), cells were incubated in Alexa-tagged secondary antibodies (Invitrogen, A31573) prepared in antibody dilution buffer for 1 h at room temperature, washed with PBS-T, stained with 1 µg/mL of 4′,6-diamidino-2-phenylindole and 0.2 µg/mL of Cell Mask Blue (Life Technologies, Invitrogen), and imaged using the microscopes, either Nikon Eclipse Ti2 or automated spinning disk confocal Opera Phenix (PerkinElmer Life Sciences). Images were analyzed by the Harmony image analysis platform or Cellprofiler (83). Harmony and Cellprofiler pipelines similar to those previously established, with appropriate modifications (84), were used. In all cases, images were segmented to identify nuclei, cells, and bacteria. NF-κB p65 nuclear:cytoplasmic ratio was assessed by comparing the NF-κB p65 fluorescence ratio between nuclear and cytoplasmic regions. Objects such as bacteria and nuclei were related to individual cells to obtain single-cell statistics, and parameters related to their numbers and intensities were extracted. MS Excel and the RStudio platform with libraries ggplot2, tidyverse, and dplyr were used for data analysis and plotting.

### Western blot

Cells were washed with ice-cold PBS on ice, scraped, and centrifuged at 1,000 rpm for 5 min at 4°C. The cell pellet was lysed with RIPA buffer (10 mM Tris chloride, 140 mM NaCl, Triton-X 100 [1%], 0.1% sodium deoxycholate, and 0.1% SDS) for 30 min on ice, followed by sonication at 4°C. Cell lysate was collected in fresh tubes after removal of cellular debris by centrifugation at 12,500 rpm at 4°C. Total protein concentration in the lysate was measured using the Bicinchoninic acid Protein Assay Kit. Sixty micrograms of protein lysate was loaded onto a 15% SDS-PAGE gel, followed by transfer to a 0.2 µm pore size polyvinylidene fluoride membrane (Bio-Rad, 16200177). The membranes were probed for the following proteins: H3K27Ac (1:2,000 dilution), AceCS1 (1:2,000 dilution from CST, D10C6), and beta-actin (1:4,000 dilution from CST, 4970S). After washing with TBST (0.001% Tween-20), blots were incubated with horseradish peroxidase-conjugated anti-rabbit (1:10,000 dilution, ThermoFisher, A-16124) secondary antibody for 1 h at room temperature. Blots were developed using Clarity Western ECL substrate reagent (Bio-Rad, 1705061). Actin was used as a loading control. ImageJ software was used to quantify the intensity of the bands, and data were normalized to the respective loading controls in each experiment.

### ChIP-seq sample preparation, library preparation, and analysis

ChIP protocol was followed as described before (85). Briefly, 40 million THP-1 MDMs were crosslinked using 1% formaldehyde for 10 min at room temperature for both conditions, infected and uninfected. Formaldehyde was quenched by 125 mM glycine for 5 min at RT with gentle rotation. After washing with cold PBS (3×), the cells were scraped and pelleted at 3,000 rpm for 5 min at 4°C. Pellet was dissolved in lysis buffer (50 μM Tris-HCl [pH 7.4], 1% SDS, 10 μM EDTA, and protease inhibitors) and sonicated for 25 cycles (30 s ON and 30 s OFF) using Bioruptor Pico (Diagenode) to obtain fragments of 500 bp. Sonicated lysate was spun to remove the insoluble debris. Supernatant was diluted 2.5 times with dilution buffer (20 mM Tris-HCl [pH 7.4], 100 mM NaCl, 2 mM EDTA, 0.5% Triton X-100, and protease inhibitors). One hundred micrograms of chromatin was taken for each IP, and 1 μg of H3K27Ac (ab4729) and Pol II (Diagenode C15200004) antibodies was added to allow binding to the complexes overnight at 4°C with gentle rocking motion. A volume of 15 μL of a 50% slurry of Protein G Dynabeads, pre-blocked with 1% BSA, was added for 4 h. The complexes were captured on a magnetic rack and washed first with wash buffer I (20 mM Tris-Cl [pH 7.4], 150 mM NaCl, 0.1% SDS, 2 mM EDTA [pH 8.0], and 1% Triton X-100) by rotation for 5 min at 4°C, then with wash buffer II (20 mM Tris-Cl [pH 7.4], 500 mM NaCl, 2 mM EDTA [pH 8.0], and 1% Triton X-100), followed by wash buffer III (10 mM Tris-Cl [pH 7.4], 250 mM LiCl, 1% NP-40, 1% sodium deoxycholate, and 1 mM EDTA [pH 8.0]), and finally with TE buffer (pH 8.0). The chromatin was then eluted in an elution buffer (100 mM sodium bicarbonate and 1% SDS) for 30 min at 37°C in a thermomixer. The supernatant was collected in separate tubes, and 14 μL of NaCl (5 M) was added to 200 μL of eluted samples and kept overnight at 65°C for de-crosslinking, followed by purification with phenol:chloroform:isoamyl alcohol. Purified chromatin was dissolved in 1× TE (pH 8) and used further for ChIP-Seq library preparation.

Library preparation was performed as per the manufacturer’s instructions for the NEBNext ChIP-seq library preparation kit (New England Biolabs). Briefly, at least 10 ng of chromatin was subjected to end repair, T-tailing, and ligation of adapters compatible with the Illumina sequencing platform. The amplified library was size-selected using AMPure XP beads to enrich fragments from 300 to 500 bp in size. Twelve picomoles of library was used for cluster generation, and sequencing was performed in 1 × 50 bp format on HiSeq 2500 (Illumina Inc.). The sequenced reads were aligned to the hg19 assembly using default Bowtie2 options. Tag directories were made from the aligned reads using the HOMER (http://homer.ucsd.edu/homer/) makeTagDirectory command. The artifacts from clonal amplification were neglected, as only one tag from each unique genomic position was considered (-tbp 1). The threshold was set at a false discovery rate (FDR) of 0.001, determined by peak finding using randomized tag positions in a genome with an effective size of 2 × 10^9^ bp. For Pol II, seed regions were initially found using a peak size of 150 bp (FDR < 0.001) to identify enriched peaks. Enriched regions separated by <1 kb were merged and considered as blocks of variable lengths. For histone marks, seed regions were initially found using a peak size of 1,000 bp (FDR < 0.001) to identify enriched loci. Enriched regions separated by <2.5 kb were merged and considered as blocks of variable lengths. HOMER make UCSC file command was used to generate bedgraph files of the read densities across the genome, and this track was uploaded to UCSC genome browser. The tag directories generated were used to quantify signal at various regions of interest using annotatePeaks.pl. DiffBind was used to perform differential ChIP-seq binding analysis.

### ChIP-qPCR

To validate enrichment of H3K27Ac at the IL-1β locus following infection with *Mtb*, ChIP DNA obtained as described above was analyzed by qPCR. The DNA pellet was resuspended in 100 µL of 1× TE buffer (Tris-EDTA, pH 8.0). Purified ChIP DNA and the corresponding input DNA were subjected to quantitative PCR using PowerUp SYBR Green Master Mix (Applied Biosystems). Each reaction was performed in technical triplicate using 4 μL of ChIP DNA in a total reaction volume of 10 μL containing 5 μL of 2× SYBR Green Master Mix and 1 μL of 10 μM of each primer. Amplification was carried out under the following cycling conditions: an initial denaturation at 95°C for 2 min, followed by 40 cycles of 95°C for 10 s and 60°C for 1 min (combined annealing and extension). ChIP enrichment was quantified using the percent input method for both antibody-immunoprecipitated samples and bead-only controls. Primers were designed to amplify ~80–150 bp regions within the promoter region identified from ChIP-seq peaks for H3K27Ac and were validated for amplification efficiency before use. For validation of enrichment at the IL-1β promoter, the following primers were used: forward primer; 5′-GAGCTCTCTGTCAATTGCAGGAG-3′; reverse primer, 5′-TAGGCAGAGCTCATCTGGCAT-3′.

### qRT-PCR

Total RNA was isolated using the TRIzol Reagent (Thermo Fisher Scientific, 15596018) according to the manufacturer’s protocol. For each condition, 1 µg of RNA was reverse-transcribed to generate cDNA using the RevertAid First Strand cDNA Synthesis Kit (Genetix, K1622). Quantitative PCR was carried out using PowerUp SYBR Master Mix (ThermoFisher Scientific, K0221) on a Bio-Rad RT-PCR with specific primer sets. The cycling conditions were as follows: 50°C for 2 min, 95°C for 10 min, followed by 40 cycles of 95°C for 15 s, 60°C for 30 s, and 72°C for 30 s. Gene expression levels were quantified relative to β-actin. Primer sequences were as follows: actin forward, 5′-ATGGAGTCCTGTGGCATCC-3′; reverse, 5′-AGGGCAGTGATCTCCTTCTG-3′; Kat8 forward, 5′-TGACCAAGACAGTGAAGGATGCT-3′; and reverse, 5′-GGATCTTGTCCACATACTTCACCT-3′.

### RNA seq—sample preparation, data acquisition, and analysis

RNA was isolated using TRIzol (Invitrogen) as per the manufacturer’s protocol. After obtaining the RNA from infected and uninfected conditions, RNA was introduced to NEBNext rRNA Depletion Kit v2 (E7400X) for depletion of rRNA from the prepared RNA sample, followed by library preparation using the library preparation kit NEBNext Ultra II Directional RNA Library Prep with Sample Purification Beads (E7765L). Prepared libraries were used to run on the HiSeq 2500-Illumina platform using 1 × 50 bp sequencing format. Furthermore, the RAW data were analyzed to find the differential expression between uninfected and infected conditions.

The raw reads were mapped to the hg19 assembly using HISAT2 in a strand-specific manner. Tag directories were made from the aligned reads using the HOMER makeTagDirectory command. HOMER make UCSC file command was used to generate strand-specific bedgraph files of the read densities across the genome, and this track was uploaded to the UCSC genome browser. DESeq2 was used to perform differential gene expression analysis. The three closest peaks of H3K27Ac to genes that were up- and downregulated by 1.5-fold with 0.05 *P*-adjusted were considered for the intersection of H3K27Ac peaks and gene expression. For analysis of publicly available data sets, FASTQ files (GEO data set GSE227851) were downloaded, and RNA-seq reads were aligned against the human reference genome *Homo sapiens* GRCh38 using HISAT2 version 2.2.1. The read counts to genes were created using featureCounts. The featureCount matrix file was loaded into IDEP 0.96. The count data were transformed using rlog to generate principal component analysis. Differential expression was determined using DESeq2 in IDEP 0.96 (86).

### Cytokine array

The RayBio C-Series Human Cytokine Antibody Array 5 Kit (AAH-CYT-5-8) was used to measure cytokine levels in conditioned media following the manufacturer’s instructions. Briefly, cytokine array membranes were blocked with the provided blocking buffer at room temperature for 30 min and incubated overnight at 4°C with 1 mL of conditioned media. Membranes were washed three times with wash buffer I and twice with wash buffer II at room temperature for 5 min per wash, followed by a 2-h incubation at room temperature with biotin-conjugated antibodies. The membranes were then washed, incubated with horseradish peroxidase-conjugated streptavidin for 2 h at room temperature, and treated with detection buffer for 2 min. Protein spots were detected using the iBright FL1500 Gel Documentation system. Spot densities corresponding to individual cytokines were quantified using Fiji according to the manufacturer’s instructions.

### Treatments

THP-1 MDMs were pre-treated with recombinant human interleukin-1Ra (anakinra, Calbiochem, 07616) at a concentration of 10 µg/mL for 16 h. Following this pretreatment, CM was added to the cells for 24 h in the presence or absence of anakinra. For all conditioned media treatments, 12% CM was used unless otherwise specified. For the SB216763 treatment, THP-1 MDMs were first infected with *Mycobacterium tuberculosis* for 4 h. After removing the extracellular bacteria, the cells were incubated with SB216763 at 10 µM for 48 h. Z-VAD(OMe)-FMK (MedChemExpress, 14463) was used at 50 µM. In the pHrodo *E. coli* experiments, THP-1 MDMs were pre-treated with cytochalasin D (Sigma-Aldrich, C8273) at a concentration of 10 µM for 1 h before incubation with pHrodo. Lipopolysaccharide (Merck, L2880) stimulation was given either at 10 ng/mL for 24 h or 1 µg/mL for 4 h. Purified human interleukin-1β (Miltenyi Biotech, 130-093-897) treatment was done at 10 ng/mL to the naive cells for 6 h.

### Phagosome maturation of pHrodo *E. coli* bioparticles by flow cytometry

RAW264.7 macrophages were pulsed with pHrodo *E. coli* particles (Invitrogen, P35361) at 20 µg/mL for 6 h, followed by analysis using FACS Fortessa (BD Biosciences). Using a forward scatter area versus side scatter area plot, the viable RAW 264.7 macrophage population was gated for further analysis. Gates for stained cells were defined based on pHrodo fluorescence levels in the unstained sample. The median fluorescence intensity of pHrodo *E. coli* particles was compared between the different experimental conditions. Analysis was performed using FlowJo software.

### Phagosome maturation of pHrodo *E. coli* bioparticles by imaging

THP-1 MDMs were stained with 1 µg/mL Hoechst-33342 (ThermoFisher Scientific, H1399) for 10 min, washed with complete RPMI, and incubated with pHrodo *E. coli* (20 µg/mL) (87). Cells were transferred to the on-stage incubator in the Nikon Eclipse Ti2 microscope and imaged at 30-min intervals for 7.5 h at 37°C and 5% CO_2_. Ten fields were recorded for each condition. For analysis, individual cells were manually segmented from bright-field images, and the intracellular fluorescence intensity of pHrodo-*E. coli* was quantified per cell at each time point and normalized to the corresponding value at the cell’s first time point. Data were plotted using RStudio (ggplot2, tidyverse, and dplyr).

### IL-1β enzyme-linked immunosorbent assay

Cell culture supernatants were harvested from THP-1 MDMs, and IL-1β levels were measured using a human IL-1β uncoated enzyme-linked immunosorbent assay kit from R&D Systems (DY201) according to the manufacturer’s instructions.

### Intracellular IL-1β staining

THP-1 MDMs were treated with 2 μg/mL brefeldin A (Sigma-Aldrich, B6542) and 2 μM monensin (Sigma-Aldrich, M5273) for 3 h. After the incubation, cells were detached gently using a cell scraper and centrifuged at 800 rpm for 5 min at 37°C. Cell pellets were washed with PBS and fixed with 4% PFA for 20 min, followed by washes with PBS. Cells were permeabilized with 0.1% Triton X-100 for 5 min at room temperature, washed with PBS, and incubated with Alexa Fluor 647 mouse anti-human IL-1β (BD Pharmingen, 567773) for 30 min on ice. After washing with ice-cold PBS, cells were resuspended in 1× PBS and immediately analyzed in a flow cytometer (BD Fortessa). Typically, 30,000 events were collected per condition. During the analysis, the region of each sample was selected based on forward scatter and side scatter parameters to exclude debris and dead cells. Infected cells were identified and gated based on the presence of the *Mtb*-mCherry signal. Data were analyzed in FlowJo and plotted in RStudio. For imaging assays, THP-1 MDMs were fixed with 4% PFA and permeabilized with Triton X-100 (0.1%) for 5 min at room temperature, followed by PBS washes. Cells were incubated overnight at 4°C with Alexa Fluor 647 mouse anti-human IL-1β antibody (5 µL/test). After washing with PBS, nuclei were stained with Hoechst, and the cytoplasm was labeled with CellMask Blue. Images were acquired using a Nikon Ti2 microscope. Cell segmentation and analysis were performed using CellProfiler. Cells were classified as *Mtb*-infected or bystander based on the presence of the *Mtb* signal. The percentage of IL-1β-positive cells was quantified in infected and bystander populations based on intracellular IL-1β signal intensity.

### Training in THP-1 MDMs

THP-1 MDMs were used for trained immunity experiments. *Mtb* infection or *Mtb* CM treatment was used as the primary stimulus for the indicated time periods, following which cells were washed with RPMI. LPS treatment (10 ng/mL) for 24 h was used as secondary stimuli, following which supernatants were collected and stored at −80°C until cytokine (IL-1β) measurement as a response readout. The time gap between the primary and secondary stimuli is considered a rest period. We have used a rest period of either 48 h or 5 days, depending on the experiment.

### *In vivo* infection and preparation of a single-cell suspension

C3HeB/FeJ mice were obtained from the Jackson Laboratory (USA, 000658) and bred in the Animal Colony Maintenance Facility, NCBS, Bangalore. Mouse infections were performed using a Glas-Col inhalation exposure system via nebulization for 30 min, according to the manufacturer’s protocol in a BSL III facility. At 24 h post-challenge, mice were sacrificed, and the total lung was homogenized in PBS. Initial bacterial loads were determined by plating serial dilutions of the homogenates on 7H11 plates supplemented with BBL Middlebrook OADC enrichment (BD Bioscience, USA), PANTA (BD Bioscience, USA), and 50 µg/mL hygromycin B. Plates were incubated at 37°C, and colonies were counted 3–4 weeks post-infection. For the experiments mentioned, an infection dose of ~50 CFU of *Mtb* was used. For the preparation of a single-cell suspension, lungs were dissected from infected mice 5 weeks post-infection. Single-cell suspensions were prepared using Miltenyi gentleMACS following the manufacturer’s instructions. Briefly, dissected lungs were transferred to a Miltenyi gentleMACS C-tube containing 2 mL of dissociation buffer (RPMI medium with 0.4 mg/mL of Liberase, Sigma-Aldrich, 5466202001) and 0.5 mg/mL of DNase (Sigma-Aldrich, 11284932), followed by the gentleMACS lung protocol 1. Samples were incubated at 37°C for 30 min with agitation, followed by the gentleMACS lung protocol 2. Lung homogenate was filtered through a 70-μm cell strainer and centrifuged at 1,200 rpm for 5 min at room temperature. The pellet was re-suspended in 1 mL of erythrocyte lysis buffer (155 mM NH_4_Cl, 12 mM NaHCO_3_, and 0.1 mM EDTA) for 1 min and immediately diluted with 10 mL of RPMI medium. After one wash with RPMI, cells were resuspended in RPMI medium supplemented with 10% FBS and plated for 2 h in a 96-well plate. Non-adherent cells were washed, and adherent cells were incubated for 16 h. Cells were fixed with 4% PFA and immunostained with anti-F4/80 antibody, a macrophage marker (Abcam, ab6640), to check for macrophage purity (see Fig. S7 at https://doi.org/10.6084/m9.figshare.32879825).

### Growth kinetics of *Mtb* by flow cytometry

For superinfection assays, THP-1 monocytes differentiated with PMA were first infected with either *Mtb*-H37Rv expressing tdTomato or *Mtb*-GFP H37Rv at an MOI of 6. The cells were incubated with bacteria for 4 h at 37°C and 5% CO_2_ to allow *Mtb* entry. After this incubation, extracellular bacteria were removed, and cells were further incubated in complete RPMI for 48 h. After 48 h, infected cells were challenged with a second round of infection, either by *Mtb*-GFP H37Rv or *Mtb*-H37Rv expressing tdTomato. Two MOIs were used: 6 (low MOI) and 12 (high MOI). At the indicated time points, cells were fixed with 4% paraformaldehyde for 20 min. The proportions of cells expressing tdTomato and GFP were assessed over time using a BD FACSAria III Flow Cytometer. Briefly, cells were scraped, and each sample was prepared in technical triplicate. Data were analyzed using FlowJo software. Thresholds for positive detection of tdTomato and GFP were set using uninfected cells as a negative control. GFP median intensities were calculated from gated populations of bystander, infected, and naive cells. For each sample, 50,000 events were acquired. Single-color control samples were collected and used for setting up gates and color compensation.

### NF-κB luciferase reporter assay

NF-κB activation was quantified using a dual-luciferase reporter assay in HeLa cells. Cells were seeded in 24-well tissue culture plates one day before transfection to achieve ~70%–80% confluency at the time of transfection. Cells were co-transfected with 0.5 μg of the NF-κB-responsive firefly luciferase reporter plasmid pGL4.32[luc2P/NF-κB-RE/Hygro] (Promega, 9PIE849) and 0.1 μg of a *Renilla* luciferase control plasmid (hRluc; Promega) using Lipofectamine 3000 (Thermo Fisher Scientific, L3000015) according to the manufacturer’s instructions. The *Renilla* plasmid served as an internal control to normalize for transfection efficiency and cell viability. Transfection complexes were prepared in serum-free medium and added to cells for 6 h, after which the medium was replaced with fresh complete DMEM medium, and cells were incubated at 37°C in a humidified CO_2_ incubator.

At 36 h post-transfection, cells were pretreated with 10 μg/mL anakinra, an IL-1 receptor antagonist, for 30 min before stimulation. Cells were then stimulated for 6 h with conditioned media collected from either uninfected or infected THP-1 MDMs. Following stimulation, cells were washed with PBS and lysed in Passive Lysis Buffer (Promega, E1960) with gentle rocking at room temperature to ensure complete lysis. Luciferase activity was measured using the Dual-Luciferase Reporter Assay System on the Multimode Reader Varioskan Lux. Firefly luciferase activity was normalized to *Renilla* luciferase activity.

### Cell death assay by flow cytometry

Cell death was quantified by flow cytometry using a fixable amine-reactive viability dye that discriminates live and dead cells based on membrane integrity. Following the second infection (day 4), THP-1 MDMs were gently harvested and washed twice with phosphate-buffered saline to remove residual media and serum components that can interfere with dye labeling. Cells were stained with Fixable Viability Stain 440UV (BD Biosciences, Cat. No. 566332) according to the manufacturer’s instructions. Briefly, cells were resuspended in incomplete RPMI (serum-free) containing the diluted viability dye and incubated for 10 min at 37°C. Following staining, cells were washed with PBS to remove excess dye and fixed with 4% paraformaldehyde for 20 min at room temperature. After fixation, cells were washed and resuspended in PBS. Samples were analyzed by flow cytometry, and debris and doublets were excluded using forward- and side-scatter parameters. Dead cells were identified based on increased fluorescence intensity of the viability dye using standard gating strategies.

## Data Availability

The NCBI accession number for ChIP and RNA seq data sets from this study is GSE278768.
